# Proteomic Dissection of Seed Germination and Seedling Establishment in *Brassica napus*

**DOI:** 10.3389/fpls.2016.01482

**Published:** 2016-10-24

**Authors:** Jianwei Gu, Hongbo Chao, Lu Gan, Liangxing Guo, Kai Zhang, Yonghong Li, Hao Wang, Nadia Raboanatahiry, Maoteng Li

**Affiliations:** ^1^Department of Biotechnology, College of Life Science and Technology, Huazhong University of Science and TechnologyWuhan, China; ^2^Hubei Collaborative Innovation Center for the Characteristic Resources Exploitation of Dabie Mountains, Huanggang Normal UniversityHuanggang, China; ^3^Hybrid Rapeseed Research Center of Shaanxi Province, Shaanxi Rapeseed Branch of National Centre for Oil Crops Genetic ImprovementYangling, China

**Keywords:** seed germination, *Brassica napus*, proteomic analysis, storage reserves

## Abstract

The success of seed germination and establishment of a normal seedling are key determinants of plant species propagation. At present, only a few studies have focused on the genetic control of seed germination by using a proteomic approach in *Brassica napus*. In the present study, the protein expression pattern of seed germination was investigated using differential fluorescence two-dimensional gel electrophoresis in *B. napus*. One hundred and thirteen differentially expressed proteins (DEPs) that were mainly involved in storage (23.4%), energy metabolism (18.9%), protein metabolism (16.2%), defense/disease (12.6%), seed maturation (11.7%), carbohydrate metabolism (4.5%), lipid metabolism (4.5%), amino acids metabolism (3.6%), cell growth/division (3.6%), and some unclear functions (2.7%) were observed by proteomic analysis. Seventeen genes corresponding to 11 DEPs were identified within or near the associated linkage disequilibrium regions related to seed germination and vigor quantitative traits reported in *B. napus* in previous studies. The expression pattern of proteins showed that heterotrophic metabolism could be activated in the process of seed germination and that the onset of defense mechanisms might start during seed germination. These findings will help generate a more in-depth understanding of the mobilization of seed storage reserves and regulation mechanisms of the germination process in *B. napus*.

## Introduction

The success of seed germination and establishment of a normal seedling are determinant features for the propagation of plant species, which are of both economic and ecological importance (Rajjou et al., 2012). Seed germination and seedling establishment depend on the mobilization of storage reserves. For oilseed crops, effective degradation of storage lipid is essential for the success of seedling establishment, which is extremely important for plant adaptation to terrestrial environments (Bewley, 1997; Pritchard et al., 2002; Graham, 2008). In recent decades, many studies have been carried out on seed germination through physiological, proteomic, or transcriptomic analysis in *Arabidopsis* (Gallardo et al., 2001, 2002; Müller et al., 2006), rice (*Oryza sativa*; Yang et al., 2007), barley (*Hordeum vulgare*; Sreenivasulu et al., 2008), maize (*Zea mays*; Guo et al., 2013), soybean (*Glycine max*; Xu et al., 2011), *Lepidium sativum* (Müller et al., 2006), *Jatropha curcas* (Yang et al., 2009), *Brassica napus* (Li et al., 2005; Ge et al., 2013), and others. These studies have provided information about many aspects of the seed germination process, such as the roles of gibberellin and abscisic acid (Gallardo et al., 2002; Reyes and Chua, 2007), radicle emergence (Guo et al., 2013), defense (Rajjou et al., 2006; Xu et al., 2011), endosperm weakening (Zhang et al., 2014), and the mobilization of energy reserves (Kelly et al., 2011; Han et al., 2013), but the detailed regulatory mechanisms in the process of seed germination are still unclear.

Seed germination is a complex process controlled by many mechanisms. Plant hormones are the key regulation factors for breaking of seed dormancy and initiation of seed germination (Bewley, 1997; Gallardo et al., 2002; Rajjou et al., 2006; Reyes and Chua, 2007; Liu et al., 2013). Mobilization of storage reserves is essential for seed germination, but different storage reserves may have different roles during seed germination, such as seed storage oil mobilization, which was indicated as important but not essential for germination or seedling establishment in *Arabidopsis* (Pinfield-Wells et al., 2005; Kelly et al., 2011). Metabolic and regulatory network models in rice and *Arabidopsis* have been constructed in previous studies (Bassel et al., 2011; He et al., 2011), which showed that different crop species might have distinct mechanisms for reserve mobilization during germination (Han et al., 2013). Rapeseed is one of the most important oil crops in the world; rapeseed oil accounts for about 13–16% of the world vegetable oil production (Hajduch et al., 2006; Obermeier et al., 2013; Wang and Yin, 2014). In addition, rapeseed is also a potential bio-energy crop to alleviate the global energy shortage (Tsadilas and Shaheen, 2013). Seed germination and vigor in *B. napus* have been investigated in recent years, and it has been revealed that these are significantly influenced by many factors, such as salt (Srivastava et al., 2004; Bybordi and Tabatabaei, 2009), temperature (Kondra et al., 1983; Zhang et al., 2015), plant hormones (Schopfer and Plachy, 1985; Nguyen et al., 2016), and aging (Zhang et al., 2006; Janmohammadi et al., 2008), and can be enhanced by priming (Zheng et al., 1994; Mohammadi, 2009; Benincasa et al., 2013; Hatzig et al., 2014). In addition, many genes (Li et al., 2005; Ge et al., 2014; Kubala et al., 2015; Nguyen et al., 2016), proteins, (Srivastava et al., 2004; Kubala et al., 2015), or quantitative trait loci (QTLs; Nagel et al., 2011; Hatzig et al., 2015) have been shown to participate in the regulation of seed germination and vigor in *B. napus*. This research is consistent with previous research in other organisms, but is still not enough to clearly elucidate the regulation mechanisms of *B. napus* seed germination.

Important candidate genes can be easily identified in complex pathways to dissect genetic control mechanisms by combining the QTL mapping method with other “-omics” methods (Long et al., 2007; Gan et al., 2013; Schiessl et al., 2014). Gan et al. (2013) compared the QTLs for oil and total protein content in the “Tapidor” × “Ningyou7” cross doubled haploid (TNDH) mapping population with candidate genes that corresponded to differentially expressed proteins (DEPs) in two *B. napus* cultivars with high and low oil content. A total of 117 candidate genes were found located in the QTL confidence intervals for oil or protein content, which indicated that these DEPs might be involved in oil or protein formation. By using a similar approach, a dozen resistance gene loci for clubroot and blackleg have been identified in *Brassica* crops (Li and McVetty, 2013), and seven candidate genes that might be important for waterlogging tolerance and were *co-localized* with the QTL identified in maize were observed (Osman et al., 2013). These results have indicated the advantage of this candidate gene mapping approach in the dissection of complex pathways and regulation mechanisms.

In this study, differential fluorescence two-dimensional gel electrophoresis (2-D DIGE) was used to analyze the DEPs in different germination processes of *B. napus*, and a metabolism regulation pathway was constructed based on the DEPs. The results provide a more in-depth understanding of the mobilization of seed storage reserves and regulation mechanisms of the germination process in *B. napus*. With the aid of QTL analysis and genome-wide association mapping, new insights for seed germination in *B. napus* could be revealed.

## Materials and methods

### Plant material

The mature seed of *B. napus* (7792-95/772/772) was provided by Shaanxi Hybrid Rapeseed Research Center. The seeds were imbibed on two layers of moist filter paper (Whatman, GE healthcare, UK) at 28°C in the dark. The whole seeds were collected at 0, 6, 12, 18, 24, 36, 48, 60, and 72 h after imbibition (HAI) and stored at −70°C until further use.

### Tissue preparation for transmission electron microscopy

The transmission electron microscopy (TEM) analysis followed the methods of Gan et al. (2013). Cotyledon tissues from dry and germinated seeds were fixed with 2.5% glutaraldehyde and postfixed in 1% OsO_4_, then dehydrated by a series of acetone. The samples were embedded in Spurr's epoxy resin and polymerized at 60°C for 24 h after infiltration through a series of acetone/Spurr's epoxy resin. The thin sections (70 nm) that were readily cut by an ultramicrotome (Leica MZ6, Germany) were collected onto copper grids, post-stained with supersaturated uranyl acetate and 0.4% lead citrate, respectively, rinsed six times, each for 15 s, with distilled water, and observed under a JEOL JEM-1230 transmission electron microscope.

### Oil content and fatty acid composition analysis

The oil content during seed germination and post-germination growth was measured using a modified method of Wei et al. (2009). Each sample was extracted simultaneously in three replicates. For fatty acid (FA) analysis, the seeds or seedling tissues were milled and then vacuum freeze-dried overnight. The FAs were then extracted using the method of Browse et al. (1986). The absolute content of FAs was determined using gas chromatography (Shimadzu GC-2010, Japan, with DEGS-diethyl glycol succinate column) referring to Browse et al. (1986) and Rücker and Röbbelen (1996). Relative content of FAs was calculated from five independent biological replicates. The difference in FA contents between different processes of germination was evaluated by one-way analysis of variance (ANOVA) test.

### Total protein and sugar analysis

Total protein was determined by using the Bradford method (Kruger, 2009). Between 10 and 100 μg protein in 100-μl total volume was collected into a test tube. The assay reagent (5 ml) was added into each tube and mixed well by inversion or gentle mixing on a vortex machine. The absorption value was then measured at 595 nm. γ-Globulin (Sigma) was used for the calibration curve, and 100 μl of distilled water was used as a blank.

To measure the total sugar content in germinating *B. napus* seed, the standard curve obtained by using glucose content was projected on the x-axis and the absorbance value on the y-axis. The sample (0.1 g) was weighed and then added to 1.5 ml water and 1 ml 6 M HCl and heated in a boiling water bath for 30 min. After the mixture was cooled down at room temperature, the pH was adjusted to neutral by using 10% NaOH solution. The filtrate was then filtered and diluted in 50 ml distilled water as sample solution. The sample solution was analyzed at an absorbance of 620 nm, and the total sugar content of samples was calculated according to the standard curve (Wen et al., 2005).

### Protein extraction and protein labeling with CyDye

Proteins were extracted using a modified protocol according to Gan et al. (2013). For each sample, about 0.5 g seeds or seedlings was ground into fine powder. About 100 mg of each sample was homogenized in 750 μl Tris-saturated phenol (pH > 7.8) and 750 μl homogenization buffer [0.1 M Tris-HCl (pH 7.5), 0.9 M sucrose, 10 mM EDTA, 0.4% (g/ml) DTT] in an ice bath for 30 min. The homogenate was centrifuged at 5000 g for 15 min at 4°C. The supernatant was transferred to a new tube and precipitated using 1.5 ml 0.1 M ammonium acetate-methanol solution at −20°C overnight. The mixture was centrifuged at 5000 g for 10 min at 4°C, and the supernatant was discarded. The precipitation was washed twice with 0.1 M ammonium acetate-methanol solution and twice with acetone, then dried at room temperature for about 5 min. The dried powder was dissolved in a buffer solution containing 7 M urea, 2 M thiourea, and 4% CHAPS (pH 8.8). Three independent biological replicates were completed for each time-point of seed germination (0, 6, 12, 18, 24, and 36 HAI).

The concentration of proteins was measured using a 2-D Quant kit (GE Healthcare, UK) according the manufacturer's instructions, and then adjusted to 5 μg/μl. For labeling of the proteins, 50 pmol CyDye was mixed with 6 μl protein sample and incubated on ice for at least 2 h in the dark. The labeling reaction was terminated by adding 1 μl 10 mM lysine (Tang et al., 2008).

### Gel electrophoresis, image scan, and data analysis

For 2-D DIGE analysis, a mixture of Cy2-, Cy3-, or Cy5-labeled protein was mixed with 2-D DIGE buffer (7 M urea, 2 M thiourea, 4% CHAPS, 0.4% DTT, 0.5% IPG buffer) and then loaded on an immobilized pH gradient (IPG; Amersham Biosciences, Uppsala, Sweden) for isoelectric focusing (Tang et al., 2008). The running conditions were as follows: rehydration for 14 h at 20°C, followed by holding at 100, 300, 600, and 1000 V for 1 h at each step; then the voltage was raised to 10,000 V linearly and held until reaching a total value of 120,000 V-h. Second-dimension electrophoresis was performed using 12.5% SDS-polyacrylamide gel (Gan et al., 2013).

The images of Cy2-, Cy3-, and Cy5-labeled proteins were acquired by a Typhoon 8600 scanner (GE Healthcare, UK) using difference wavelength and analyzed by DeCyder 6.5 software (GE Healthcare, UK). Spot detection was performed by the DIA (differential in-gel analysis) module. After removing the artifact spots by manual editing, the images were further analyzed by the DeCyder BVA (biological variation analysis) module. For each treatment, images from at least three biological replicates were used for statistical analysis.

### Protein spot picking and identification

The preparation of isoelectric focusing with 1 mg protein sample loaded into IPG strips for spot picking and second-dimension electrophoresis were performed as mentioned above. Gels were stained with CBB R-250 according to the protocol of Gan et al. (2013). The stained gels were scanned by a UMAX Power Look 2100XL scanner (UMAX, Inc., Taipei, China). These images were matched with the DIGE images to identify spots of interest. Protein spots were manually excised from the gels and cut into small pieces.

Protein spots were digested according to the method of Katayama et al. (2001). The digested protein samples were subjected to MS and MS/MS analysis by using an ABI 4800 MALDI-TOF/TOF Plus mass spectrometer (Shu et al., 2011). Both the MS and MS/MS data were integrated and processed by the GPS Explorer V3.6 software (Applied Biosystems) with default parameters. For protein identification, the acquired MS/MS spectra were automatically searched on the NCBInr green plants database, using the MASCOT V2.1 search engine (Matrix Science, London, UK). Search parameters were set as taxonomy: Rosids; enzyme specificity considered: trypsin; max missed cleavages: 1; fixed modifications: carbamidomethyl (C); variable modifications: Acetyl (Protein N-term), Deamidated (NQ), Dioxidation (W), and Oxidation (M); peptide mass tolerance: ±100 ppm; fragment mass tolerance: ±0.5 Da.

To minimize the inclusion of false positive hits, matches to peptides identified by SEQUEST were filtered according to their charge state, cross-correlation score (Xcorr), and normalized difference in correlation score (deltaCn). Peptide hits were accepted when singly, doubly, and triply charged peptides were with Xcorr >1.9, 2.2, and 3.75, respectively, and deltaCn >0.1 in all cases. After the peptide sequence raw data was searched using SEQUEST, a number of other criteria were considered in the final assignment of peptide and protein identifications: the number of matching peptides, the coverage, the Xcorr, and the molecular mass and isoelectric point of the protein.

### Protein classification and hierarchical clustering analysis

Protein functional domains were predicted using the PSI and PHI-BLAST programs (http://www.ncbi.nlm.nih.gov/BLAST/). Proteins were classified into different categories by combining BLAST alignments with Gene Ontology and knowledge from the literature. A hierarchical cluster was constructed by SPSS using the K-means clustering approach in order to visualize the expression characteristics of the DEPs (Li et al., 2005).

### Mapping identification of DEPs

A genome-wide association study to define genomic regions influencing seed germination and early seedling growth was carried out by Hatzig et al. (2015). Twenty-four associated linkage disequilibrium (LD) regions were identified to be associated with seven traits. Twenty associated LD regions related to seed germination and vigor were selected for comparative study. The related traits were as follows: volume increase within first 8 h (VI), total germination rate within 72 h after initiation of imbibition (GR72), first germination time (FG), radicle elongation speed (ES), time to reach 50% of germination (T50), and germination rate within 36 h after the initiation of imbibition (GR36). To identify the DEPs comprehensively and effectively, the genes within the flanking regions up to 1000 kbp on either side of the associated LD regions were considered as candidate regions for searching DEP genes.

### Gene expression analysis of DEPs

Total RNA was extracted from 0.1 g of the frozen samples by using a RNA prep Pure Plant Kit (TOYOBO, DP441). The first chain was synthesized by ReverTra Ace qPCR RT Master Mix with gDNA Remover (TOYOBO, FSQ-301) according to the manufacturer's instructions. Expression analysis was assayed with a SYBR premix EX TaqTM kit (TaKaRa, Japan) on an ABI 7900HT Fast Real-Time PCR System. Relative gene expression was analyzed according to the method of Pfaffl et al. (2004). *Actin 2.1* was used as a reference gene (Kubala et al., 2015), and other primers used in this study are listed in Table S1. Intra-assay variation was evaluated by calculating SD errors of arithmetic means of three sample replicates.

## Results

### Seed germination process, change of major storage reserves, and FA compositions

**Figure 1:**
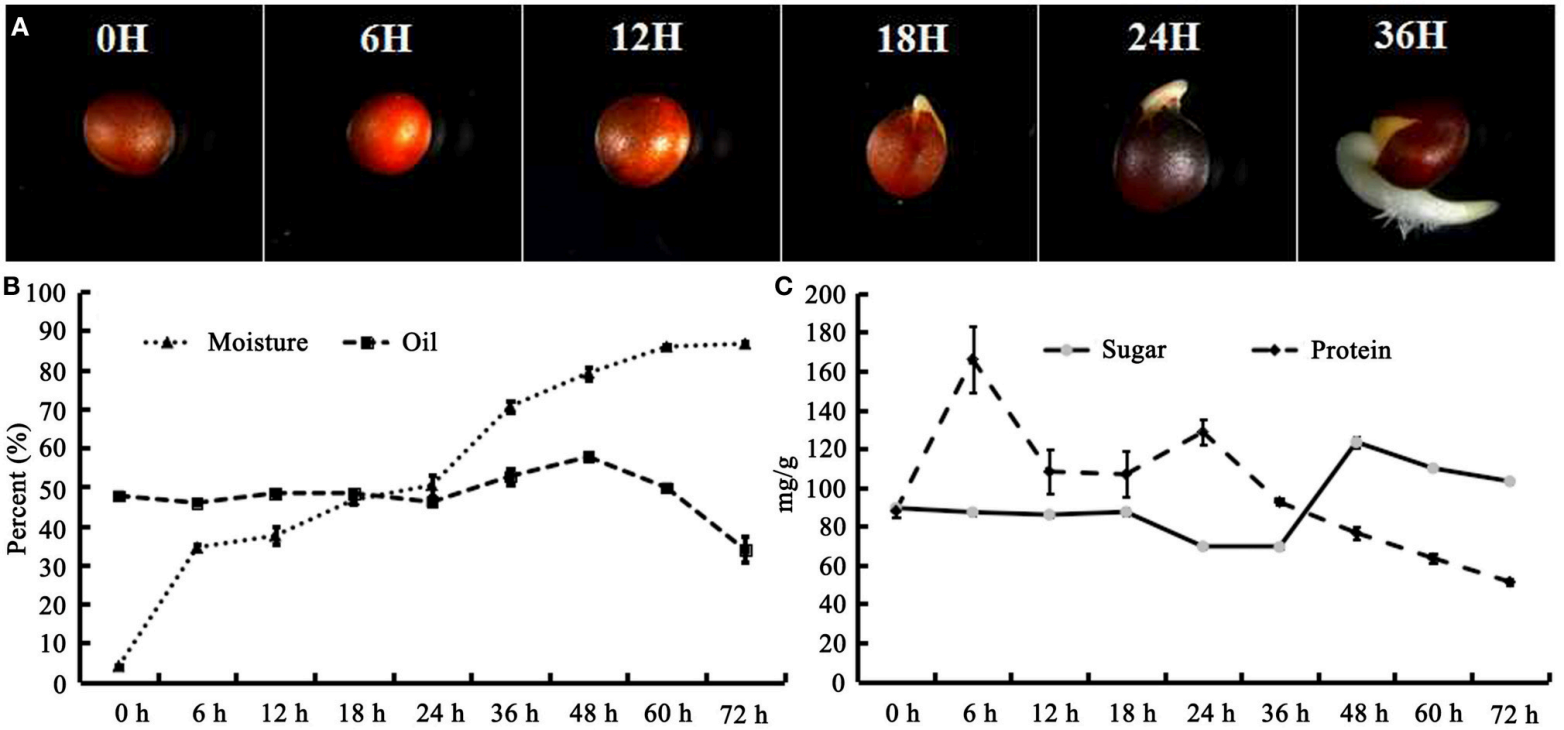
*****B. napus*** seed germination process and change of moisture, oil, protein, and sugar during seed germination and seedling establishment**. **(A)** *B. napus* seed germination. The seeds were imbibed in moisture at 28°C under dark conditions. Photographs were taken at 0, 6, 12, 18, 24, and 36 HAI; **(B)** Change of moisture and oil during germination; **(C)** Change of protein and sugar during germination. Values are means of three biological replicates (±SD).

The seeds of *B. napus* were imbibed on moist filter paper for germination under darkness. It was revealed that the seed coat was ruptured, and hypocotyl elongation and radicle protrusion were observed at 18 HAI (Figure 1A); this process could be termed germination. Subsequently, the hypocotyl and radicle continued elongation to push the cotyledon out of the seed coat (Figure 1A). To obtain an overview, post-germination growth until 72 HAI was included in this work. The moisture content increased remarkably from 3.94 to 39% during the first 6 HAI, then experienced a stage of slow increase to 50.43% until 24 HAI, and increased rapidly again to 85.96% at 60 HAI (Figure 1B).

The oil content of *B. napus* seeds was stable at the germination stage and decreased sharply with more than 48 HAI (Figure 1B). Compared with 47.68% in dry mature seeds, the oil content was decreased to 33.86% at 72 HAI (Figure 1B), indicating that the oil might have been mobilized and consumed during the germination and post-germination processes. The FA compositions of the crude oil from different stages of germinating seeds were also determined (Table 1). It was revealed that the relative contents of arachidic acid (C_20:0_) and *cis*-11-eicosenoic acid (C_20:1_) were decreased from 0.27 and 0.5% to 0.14 and 0.39% of total FAs at 6 HAI, respectively (*p* < 0.01), and even lower at 72 HAI. No drastic change was observed for other FAs, while the absolute content of total FA increased to >250 μg/mg at 36 HAI and then decreased to about 200 μg/mg at 72 HAI. A similar pattern of change was observed in the absolute content of each FA except C_20:0_ and C_20:1_, which were significantly decreased at 6 HAI and remained lower even until 72 HAI (Figure S1).

**Table 1:** **Change of FA contents for seed oil during germination and seedling establishment**.

	**Samples**								
**Fatty acid (%)^a^**	**0 h**	**6 h**	**12 h**	**18 h**	**24 h**	**36 h**	**48 h**	**60 h**	**72 h**
C16:0^c^	4.39 ± 0.05^b^	4.63 ± 0.16	4.78 ± 0.09	4.85 ± 0.07	4.76 ± 0.13	4.95 ± 0.22	5.36 ± 0.15	5.34 ± 0.43	5.92 ± 0.16
C16:1	0.16 ± 0	0.17 ± 0.01	0.18 ± 0	0.18 ± 0	0.18 ± 0	0.2 ± 0.01	0.24 ± 0.01	0.25 ± 0.02	0.3 ± 0.01
C18:0	1.23 ± 0.03	1.22 ± 0.04	1.25 ± 0.02	1.25 ± 0.02	1.12 ± 0.02	1.11 ± 0.03	1.31 ± 0.04	1.27 ± 0.13	1.29 ± 0.05
C18:1	59.8 ± 1.91	60.22 ± 3.36	60.52 ± 1.12	60.19 ± 1.29	59.33 ± 1.6	58.1 ± 2.29	61.08 ± 2.19	60.83 ± 5.75	60.57 ± 2.69
C18:2	24.69 ± 0.78	24.33 ± 1.52	24.04 ± 0.53	24.21 ± 0.54	24.75 ± 0.72	25.37 ± 1.25	26.42 ± 0.98	26.21 ± 2.08	27.08 ± 1.01
C18:3	8.88 ± 0.29	8.84 ± 0.65	8.74 ± 0.27	8.83 ± 0.25	9.42 ± 0.29	9.61 ± 0.5	10.4 ± 0.48	10.68 ± 0.77	11.27 ± 0.41
C20:0	0.27 ± 0.02	0.14 ± 0.05	0.11 ± 0.01	0.1 ± 0.00	0.09 ± 0.00	0.1 ± 0.00	0.13 ± 0.03	0.11 ± 0.03	0.11 ± 0.03
C20:1	0.5 ± 0.03	0.39 ± 0.05	0.34 ± 0.01	0.33 ± 0.02	0.3 ± 0.01	0.3 ± 0.01	0.34 ± 0.06	0.27 ± 0.03	0.3 ± 0.03
C20:2	0.04 ± 0.00	0.03 ± 0.01	0.03 ± 0.00	0.03 ± 0	0.03 ± 0.00	0.03 ± 0.00	0.05 ± 0.01	0.06 ± 0.01	0.06 ± 0.01
C22:1	0.02 ± 0.00	0.02 ± 0.01	0.01 ± 0.01	0.02 ± 0.01	0.003 ± 0.00	0.01 ± 0.01	0.01 ± 0.01	0.005 ± 0.00	0.02 ± 0.01
C22:2	0.02 ± 0.00	0.02 ± 0.00	0.02 ± 0.00	0.02 ± 0	0.01 ± 0.00	0.02 ± 0.00	0.02 ± 0.01	0.02 ± 0.01	0.02 ± 0.00

a The relative content of each FA's composition.

b Content of each FA was calculated as the percentage that each FA represented in the total measured fatty acids. Each value is the mean of five biological replicates (±SD).

c The numbers denote the number of carbons and double bonds. For example, C18:1 stands for 18 carbons and one double bond.

The total protein and sugar in germinating *B. napus* seeds was also measured (Figure 1C). Generally, the total protein increased to its highest level at 6 HAI (166.02 mg/g) and then decreased continuously to 51.31 mg/g after 24 HAI. The total sugar content remained stable before 18 HAI and then decreased to 69.27 mg/g at 36 HAI. The total sugar content was observed to sharply increase to 123.35 mg/g at 48 HAI and then slowly decrease to 103.1 mg/g until 72 HAI. These results suggested that various physiological and biochemical processes occurred during post-germination.

### Structural characteristics of germinating seed of *B. napus*

**Figure 2:**
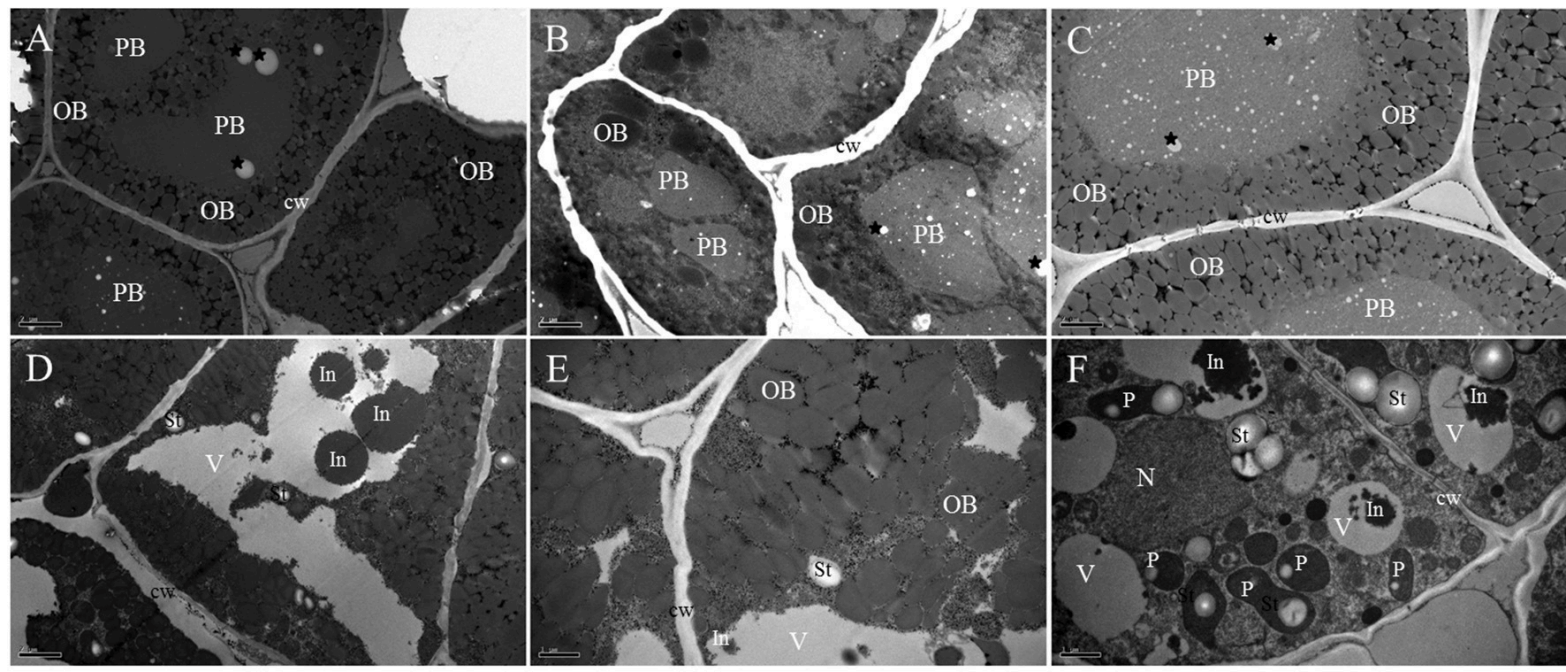
**TEM study of cotyledons during imbibition and seedling establishment. (A)** Dry mature seed. Electron-dense protein body (PB) with a homogenous matrix embedded in a cytoplasm, densely populated by small and less electron-dense OBs. Electron-light, small, rounded areas are presented in the PB matrix (black stars). **(B)** Twelve hours of germination. The cytoplasm is filled up with electron-dense PBs and numerous electron-light OBs. The different size electron-light, rounded area now is all over its matrix (black stars). **(C)** Twenty-four hours of germination. Enlarged PBs with different degrees of electron density are surrounded by OBs in close contact. The presence of electron-light, rounded area through its matrix (black stars) still can be seen. **(D)** Overall presentation of the 2 days of germination. The PB compartment is now similar to a vacuole (V) with a low electron density lumen that contains irregular electron-dense inclusions (In); in the surrounding cytoplasm, minute OBs and starch grains (St) are present. **(E)** Enlarged presentation of the 2 days of germination. The gap between the OBs is increased and boundaries of the OBs become blurred. Some cytoplasmic-like structures appear in the space between the OBs. **(F)** Three days of germination. The vacuolar compartment (V) with a low electron density lumen contains irregular, electron-dense inclusions (In); in the peripheral cytoplasm, developing plastids (P) with inner membrane system and starch grains (St) are present. A nearly rounded nucleus (N) with condensed chromatin is attached to the nuclear envelope. PB, protein body; OB, oil body; V, vacuole; St, starch grains; P, plastids; cw, cell wall. Bar **(A–D)** = 2 μm. Bar **(E,F)** = 1 μm.

TEM results allowed us to obtain additional data about the subcellular organization of the cotyledon cells during *B. napus* seed germination. In the mature dry seed, the cotyledon cells were completely filled by protein storage vacuoles (PBs) and oil bodies (OBs; Figure 2A). PBs of the cotyledon cells sh2owed different shapes (round and regular shape) with undulating contours and were formed by a homogenous and electron-dense matrix, and some electron-light, small, rounded areas were observed in the PB matrix. The rest of the cotyledon cell was filled up with smaller and electron-transparent bodies of rounded shape, corresponding to OBs (Figure 2A). No major changes were observed in cells examined before 12 HAI except for the different sizes of electron-light rounded areas in PBs, which obviously were all over the matrix (black stars; Figure 2B).

By 24 HAI, the size of the PBs was increased, and they became rounded and less electron-dense (Figure 2C), indicating that the storage protein might begin to mobilize at this stage. The small, electron-transparent, rounded area inside the PBs was still present (Figure 2C, black stars). The OBs were observed surrounding the surface of the PBs and at the cell periphery. After 2 days of germination, the PB compartment was similar to a vacuole with a low electron density lumen that contained irregular, electron-dense inclusions. OBs as well as some starch grains were observed in the surrounding cytoplasm (Figure 2D). At the same time, the gap between the OBs was increased and the boundaries of the OBs became blurred, which indicated that the storage oil was also mobilized (Figure 2E). After 3 days of growth, developing plastids were easily identified in the cytoplasm, showing the presence of starch grains and an inner membrane system (Figure 2F). Some irregular, highly electron-dense inclusions located in the vacuole-like organelles were also observed. In the cytoplasm, a nearly rounded nucleus with condensed chromatin was attached to the nuclear envelope.

### Identification of DEPs and their functional classification

**Figure 3:**
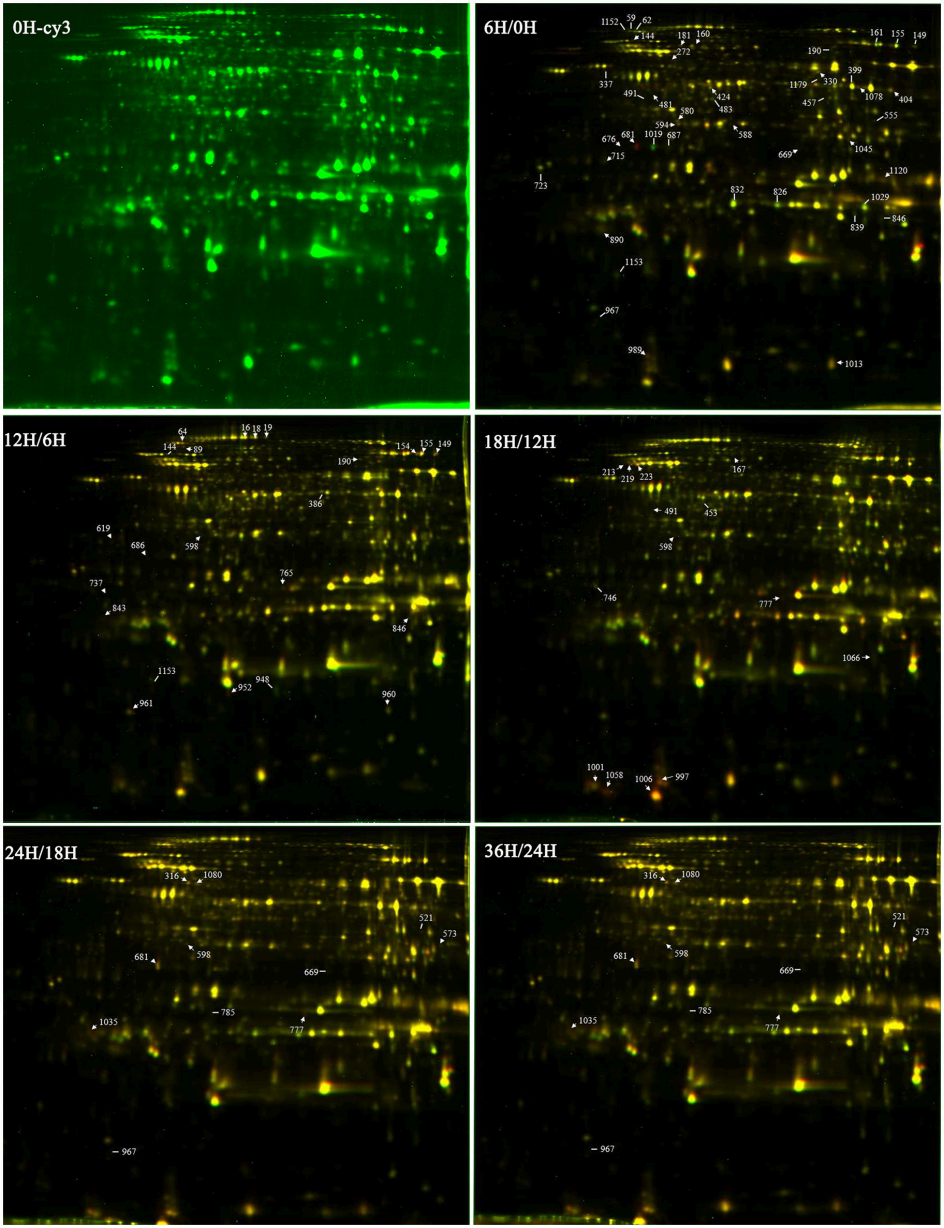
**The 2-DIGE maps of different germination stages**. 0H-cy3 represents the sample labeled with cy3. 6H/0H, 12H/6H, 18H/12H, 24H/18H, and 36H/24H represent comparisons between two samples.

**Figure 4:**
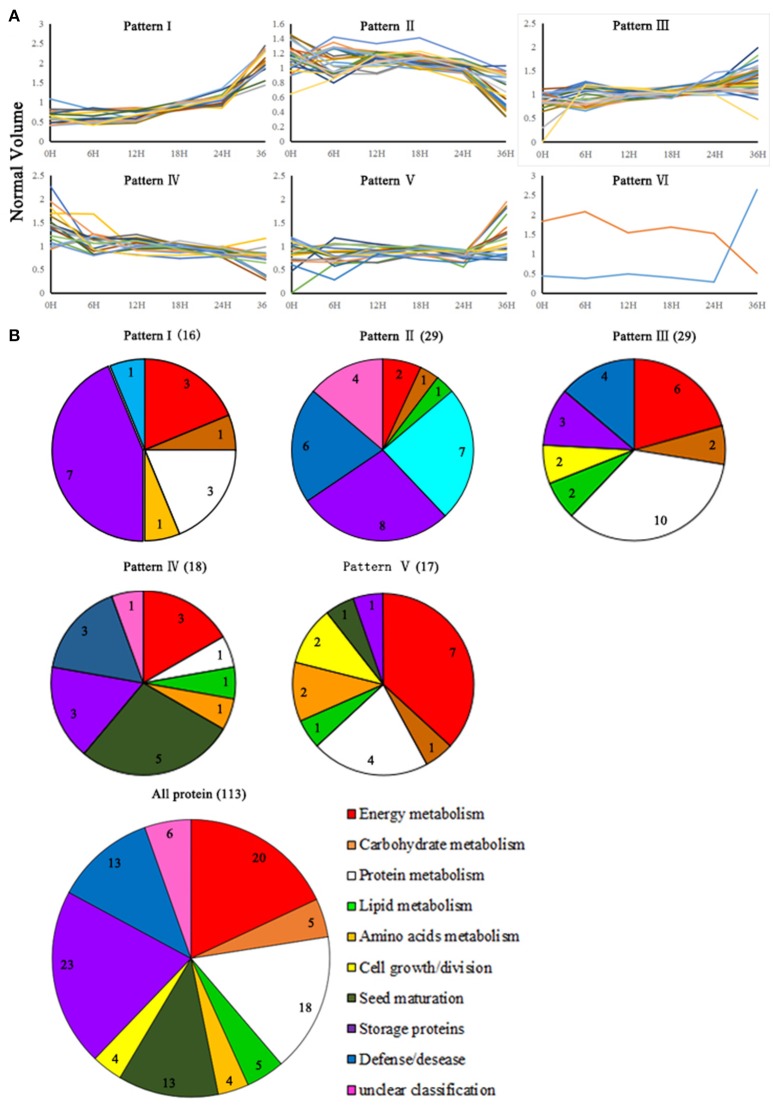
**Functional categorization and kinetic patterns of accumulation of DEPs detected in the germination process**. **(A)** The kinetic patterns of accumulation of DEPs. Patterns I–V represents different accumulation patterns groups. **(B)** The functional categorization of DEPs. Patterns I–V correspond to the functional categorization of the DEPs in different accumulation patterns groups. All protein represents the functional categorization of all DEPs.

DEPs that existed during the five seed germination stages were detected by 2-D DIGE with a pH 4–7 IPG strip. The results revealed that approximately 1300 protein spots were detected on each gel (Figure 3; Figure S2). Among the DEPs, a total of 113 protein spots were identified through MS and MS/MS analysis and searching of the Mascot database in *B. napus*. Based on Gene Ontology, BLAST alignments, and information from the TAIR and Kyoto Encyclopedia of Genes and Genomes (KEGG) databases, the 113 protein spots were classified into 10 functional categories: storage proteins (23.4%), energy metabolism (18.9%), protein metabolism (16.2%), defense/disease (12.6%), seed maturation (11.7%), carbohydrate metabolism (4.5%), lipid metabolism (4.5%), amino acids metabolism (3.6%), cell growth/division (3.6%), and some unclear proteins (2.7%; Table 2; Figure 4B). These 113 protein spots only matched 68 unique proteins in *Arabidopsis*, and 20 proteins were matched with multiple protein spots, which were associated with defense/disease, storage proteins, protein metabolism, energy metabolism, lipid metabolism, seed maturation proteins, and carbohydrate metabolism (Table 2; Tables S2–S4). These multiply matched protein spots might be generated from alternative splicing, protein metabolism, and various post-translational modifications. There were 50, 23, 14, 11, and 55 DEPs showing significantly different expression at 6, 12, 18, 24, and 36 HAI, respectively (Figure S3; Table S2). The main metabolism pathways were quite different during seed germination and seedling establishment. For instance, most of the 50 DEPs were involved in energy metabolism (20%), protein metabolism (20%), storage proteins (14%), defense/disease (10%), and seed maturation (8%) at 6 HAI, which indicated the activity of protein synthesis, processing and selective degradation, storage reserve mobilization, and the onset of defense systems at the early germination stage. While at 36 HAI, storage proteins (27.3%), energy metabolism (18.2%), seed maturation (18.2%), and protein metabolism (16.4%) related DEPs were the major significantly differently expressed DEPs (Figure S3).

**Table 2:** **Identification of DEPs from seeds during germination and seedling establishment**.

**Spot no**.	**Score^a^**	**MP^b^**	***Arabidopsis thaliana* Protein name**	**T.MW^c^**	**T.Pi^d^**	**M.Mw^e^**	**Protein ID**
**ENERGY METABOLISM**							
89	554	9 (4)	Pyruvate, phosphate di-kinase 1	103923.41	5.62	104,541	gi|674942926
167	290	6 (2)	Transketolase	79477.47	5.88	79,884	gi|674864333
181	440	8 (4)	Transketolase	79514.84	5.9	79,864	gi|674891569
260	164	4 (2)	2,3-bisphosphoglycerate-independent phosphoglycerate mutase 1	60634.86	5.42	60,825	gi|257627865
282	67	2 (1)	Phosphoglycerate mutase, 2,3-bisphosphoglycerate-independent	61011.24	5.55	61,144	gi|674896942
386	607	9 (6)	Ribulose-1,5-bisphosphate carboxylase/oxygenase large subunit	52956.06	5.87	53,436	gi|383930435
447	527	8 (4)	ATP synthase CF1 beta subunit (chloroplast)	53716.61	5.2	53,740	gi|75336517
563	415	8 (7)	Alcohol dehydrogenase 1	41260.07	5.89	41,975	gi|674954067
481	159	4 (2)	Enolase 1	51429.39	5.78	51,796	gi|674937089
1140	932	8 (7)	Bifunctional enolase 2/transcriptional activator	46107.33	5.39	46,364	gi|674868679
521	119	5 (2)	Alcohol dehydrogenase class-3	40753.85	6.51	41,583	gi|218336129
588	411	7 (5)	Phosphoglycerate kinase	42171.76	5.58	42,202	gi|259662425
594	948	9 (8)	Phosphoglycerate kinase	42171.8	5.71	42,202	gi|923669817
597	50	3 (1)	Transaldolase-like protein	50810.93	8.89	50,951	gi|674902947
669	94	2 (1)	Malate dehydrogenase 1	35711.26	8.81	35,860	gi|899226
692	275	4 (4)	Malate dehydrogenase 1	35625.23	8.54	35,831	gi|674909041
1045	157	3 (2)	Malate dehydrogenase	35718.2	6.11	36,038	gi|433335660
1179	485	9 (3)	Aldehyde dehydrogenase 2B4	58526.77	7.59	58,832	gi|674895930
573	119	2 (2)	Formate dehydrogenase	42336.4	6.81	42,595	gi|567173987
576	249	9 (5)	Adenosine kinase 2	37609.8	5.15	37,985	gi|674922784
580	851	8 (7)	Adenosine kinase 1	37697	5.38	38,129	gi|674910983
**CARBOHYDRATE METABOLIC**							
424	754	8 (6)	UDP-GLUCOSE PYROPHOSPHORYLASE 1	79239.24	5.86	79,418	gi|674889563
453	928	9 (8)	UDP-GLUCOSE PYROPHOSPHORYLASE 1	79239.24	5.86	79,418	gi|674889563
491	566	8 (4)	Glucose-1-phosphate adenylyltransferase small subunit	57044.89	5.86	57,294	gi|17865468
619	42	3 (1)	pfkB-like carbohydrate kinase family protein	41471.32	5.54	41,953	gi|674876646
523	116	6 (3)	UDP-D-apiose/UDP-D-xylose synthase 2	43928.23	5.8	44,357	gi|674900114
**PROTEIN METABOLISM**							
144	387	8 (2)	Nudix hydrolase 3	87528.24	5.08	87,873	gi|674954216
213	399	6 (3)	Heat shock protein 70-4	71101.45	5.13	71,456	gi|923515814
219	727	8 (6)	Heat shock cognate protein 70-1	70774.32	5.07	71,129	gi|2655420
223	856	8 (8)	Heat shock cognate protein 70-1	70774.32	5.07	71,129	gi|2655420
272	667	9 (6)	Heat-shock protein 70T-2	60545.43	5.38	61,020	gi|674921074
316	906	10 (10)	Heat shock protein 60	61316.63	5.64	61,620	gi|674938562
317	78	3 (2)	chaperonin 60 subunit beta 1	62472.87	6.56	62,776	gi|134104
330	185	4 (1)	T-complex protein 1 subunit alpha	59183.31	5.92	594,431	gi|674890539
337	701	10 (6)	Protein disulfide isomerase-like 1-2	55740.96	5	55,934	gi|674944991
483	633	9 (7)	Translational initiation factor 4A-1	46713.5	5.55	46,969	gi|923797802
489	83	3 (1)	Aha1 domain-containing protein	39015.31	5.71	39,162	gi|674932474
676	320	4 (4)	60S acidic ribosomal protein P0-2	34333.48	5.04	34,369	gi|674879004
681	674	4 (4)	60S acidic ribosomal protein P0-2	34333.48	5.04	34,369	gi|674879004
714	721	7 (6)	Aspartic proteinase A1	65970.86	5.04	66,727	gi|674898012
715	797	8 (8)	Aspartic proteinase A1	64115.18	5.79	64,873	gi|674901714
846	184	3 (2)	Protein-l-isoaspartate methyltransferase 2	13555.49	6.41	13,547	gi|674870345
948	359	4 (4)	Eukaryotic translation initiation factor 5A-2	17097.23	5.71	17,315	gi|40805177
1080	373	7 (3)	Heat shock protein 60-2	62918.5	5.98	63,278	gi|674913703
**LIPID METABOLISM**							
572	462	7 (5)	GDSL esterase/lipase	42700.74	6.52	43,129	gi|674877392
160	132	4 (1)	Acetyl-coenzyme A carboxylase carboxyl transferase subunit alpha	85330.2	5.58	85,391	gi|224814588
457	158	3 (1)	Beta-ketoacyl-[acyl carrier protein] synthase I	50351.14	7.99	50,890	gi|923831799
687	149	2 (1)	Enoyl-[acyl-carrier-protein] reductase [NADH]	40478.95	8.78	40,625	gi|461461
1019	485	6 (5)	enoyl-[acyl-carrier-protein] reductase [NADH]	40709.23	8.53	40,912	gi|14422259
**AMINO ACIDS METABOLISM**							
555	556	8 (6)	Glutamate dehydrogenase 1	44624.96	6.1	44,882	gi|674894393
598	453	6 (5)	Glutamine synthetase 1;1	39163.2	5.28	39,367	gi|599656
634	402	6 (6)	Spermidine synthase 1	36284.24	4.73	36,717	gi|219930913
686	486	8 (7)	Mercaptopyruvate sulfurtransferase 1	41511.74	5.89	41,714	gi|674965759
**CELL GROWTH/DIVISION**							
59	380	5 (4)	Cell division control protein 48C	93617.36	5.37	94,357	gi|923539107
62	483	7 (4)	Cell division control protein 48C	93617.36	5.37	94,357	gi|923539107
64	413	5 (4)	Cell division control protein 48C	93617.36	5.37	94,357	gi|923539107
1152	150	4 (0)	Cell division control protein 48-B	90340.5	5.04	91,082	gi|923629169
**SEED MATURATION**							
149	190	3 (3)	Late embryogenesis abundant domain-containing protein	50377.23	6.16	50,347	gi|674884534
154	147	3 (2)	Late embryogenesis abundant domain-containing protein	50377.23	6.16	50,347	gi|674884534
155	244	4 (3)	Late embryogenesis abundant domain-containing protein	50377.23	6.16	50,347	gi|674884534
161	171	3 (2)	Late embryogenesis abundant domain-containing protein	50377.23	6.16	50,347	gi|674884534
698	349	5 (4)	Seed maturation protein	26861.7	4.71	26,845	gi|674922898
704	410	8 (6)	Seed maturation protein	26682.47	4.76	26,666	gi|674894509
711	292	7 (4)	Seed maturation protein	26680.53	4.71	26,664	gi|674922578
723	428	6 (4)	Seed maturation protein	26680.53	4.71	26,664	gi|674922578
737	709	6 (6)	Seed maturation protein	26434.38	4.93	26,418	gi|674908838
746	790	7 (7)	Seed maturation protein	26525.47	5.01	26,509	gi|674940292
985	88	2 (1)	Em-like protein GEA6	9587.35	5.88	9582	gi|674908138
986	86	2 (1)	Em-like protein GEA6	9218.89	6.74	9214	gi|674878362
1025	315	6 (5)	Uncharacterized protein	27256.31	5.79	27,410	gi|674885273
**STORAGE PROTEINS**							
404	299	4 (4)	12S seed storage protein CRA1	53758.09	6.84	54,010	gi|117527
466	136	3 (2)	12S seed storage protein CRA1	12007.42	9.3	12,000	gi|17803
745	184	3 (3)	Cruciferin 3	56502.59	7.64	56,867	gi|17801
777	581	8 (6)	Cruciferin 2	52234.19	7.66	52,600	gi|674943446
798	107	4 (1)	Cruciferin 2	52234.19	7.66	52600	gi|674943446
799	219	7 (7)	Cruciferin 2	52234.19	7.66	52,600	gi|674943446
806	193	6 (6)	Cruciferin 2	51376.91	7.7	51,630	gi|461841
843	293	5 (3)	Cupin domain-containing protein	52119.45	6	52,314	gi|674928869
890	283	4 (3)	Cupin family protein	61958.72	5.52	62,489	gi|674908237
909	273	3 (3)	12S seed storage protein CRA1	12007.42	9.3	12,000	gi|17803
922	420	4 (4)	12S seed storage protein CRA1	12007.42	9.3	12,000	gi|17803
952	361	6 (5)	12S seed storage protein CRD	49151.47	5.91	49,349	gi|674923348
989	454	6 (6)	Cruciferin 3	56502.59	7.64	56,867	gi|461840
997	310	5 (5)	Cruciferin 3	54383.48	8.13	54,749	gi|12751302
1001	327	5 (5)	Cruciferin 3	56502.59	7.64	56,867	gi|461840
1006	362	5 (5)	Cruciferin 2	52234.19	7.66	52,600	gi|674943446
1013	206	4 (3)	12S seed storage protein CRA1	53742.13	6.84	53,994	gi|167134
1027	161	5 (5)	Cruciferin 2	51376.91	7.7	51,630	gi|461841
1058	220	3 (3)	Cruciferin 3	56502.59	7.64	56,867	gi|461840
1066	487	4 (4)	12S seed storage protein CRA1	12007.42	9.3	12,000	gi|17803
1120	273	4 (4)	12S seed storage protein CRA1	53742.13	6.84	53,994	gi|167134
1153	308	5 (3)	12S seed storage protein CRD	54789	6.65	55,439	gi|674900495
1182	151	2 (2)	12S seed storage protein CRA1	12007.42	9.3	12,000	gi|17803
706	425	3 (3)	Protein PAP85	54649.62	6.68	54,786	gi|674876933
740	191	2 (2)	Protein PAP85	54678.66	6.56	54,815	gi|674898689
1035	301	5 (3)	Cupin domain-containing protein	52119.45	6	52,314	gi|674928869
**DEFENSE/DESEASE**							
16	707	10 (9)	Myrosinase-binding protein 2	99463.68	5.48	99,404	gi|1655824
18	860	10 (10)	Myrosinase-binding protein 2	99463.68	5.48	99,404	gi|1655824
19	818	10 (10)	Myrosinase-binding protein 2	99463.68	5.48	99,404	gi|1655824
492	66	4 (1)	Myrosinase-binding protein 2	35586.5	5.8	35,565	gi|1655828
826	489	5 (5)	1-Cys peroxiredoxin PER1	23912.47	5.97	24,068	gi|7381260
832	519	5 (5)	1-Cys peroxiredoxin PER1	23912.47	5.97	24,068	gi|7381260
839	171	3 (2)	Glutathione S-transferase TAU 19	25657.71	6.36	25,812	gi|674954140
931	181	4 (4)	Glutathione peroxidase 6	25275.82	9.21	25,431	gi|674907835
960	689	8 (7)	Adenine nucleotide alpha hydrolases-like superfamily protein	17651.53	5.94	17,754	gi|674925789
961	609	7 (7)	Peptide methionine sulfoxide reductase B5	15623.4	4.96	15,956	gi|674911859
967	481	7 (5)	Peptide methionine sulfoxide reductase B5	15623.4	4.96	15,956	gi|674911859
1029	443	5 (5)	1-Cys peroxiredoxin PER1	23912.47	5.97	24,068	gi|7381260
399	786	8 (8)	Jacalin-like lectin domain-containing protein	48283.51	5.99	48,311	gi|674873970
1078	234	3 (2)	Jacalin-like lectin domain-containing protein	48283.51	5.99	49,249	gi|674873970
**UNCLEAR FUNCTIONS**							
190	244	4 (3)	CAP160 protein	58663.26	5.85	58,628	gi|674940618
765	637	9 (7)	Uncharacterized protein	27766.91	5.85	28,091	gi|674893407
785	534	7 (4)	Uncharacterized protein	26742.37	5.62	26,953	gi|674906795

a Protein match score in the result of mascot search.

b Number of peptides that matched in mascot search (the number of peptides which matched in mascot search and its expect value was lower than 0.05).

c,d Theoretical values for isoelectric point and molecular weight, respectively.

e Experimental molecular mass.

Eighteen DEPs involved in protein synthesis, folding, and degradation were also observed. Most of these proteins had up-regulated expression during seed germination, for example, translational initiation factor 4A-1 (spot 483), chaperonin 60 subunit beta 1 (spot 317), 60S acidic ribosomal protein P0-2 (spots 676 and 681), heat shock cognate protein 70-1 (spots 219 and 223), and T-complex protein 1 subunit alpha (spot 330) were all up regulated at the early stage of seed germination and retained relatively high expression levels during the following seed germination and post-germination seedling growth (Table 2; Table S2); this suggested that the activities of protein synthesis, processing, and selective degradation were essential for seed germination and post-germination seedling growth. Besides, 19 and 7 DEPs participated in energy and carbohydrate metabolism, such as glycolysis/gluconeogenesis, tricarboxylic acid (TCA) cycle, pentose phosphate pathway, ATP metabolism, and glycometabolism (Table 2; Table S2), most of which were also up-regulated in seed germination. For instance, malate dehydrogenase (spots 669, 692, and 1045) was found to be at low or undetectable levels in dry seeds but significantly increased in germinating seeds (Table 2; Table S2), indicating that mobilization and metabolism of the stored reserves took place in germinating seeds. Moreover, nine DEPs in other metabolism processes were observed, including lipid metabolism and amino acid metabolism. For instance, the acetyl-coenzyme A carboxylase carboxyl transferase subunit alpha (spot 160) was significantly induced from the beginning of germination, and the highest expression was observed at 36 HAI (Table 2; Table S2). Moreover, the expression of three amino acid metabolism-related DEPs, i.e., glutamine synthetase 1;1 (spot 598), spermidine synthase 1 (spot 634), and mercaptopyruvate sulfurtransferase 1 (spot 686), was stable at the beginning of germination, then increased from 12 HAI, and the highest expression level was observed at 36 HAI (Table 2; Table S2), indicating that other metabolic processes were also changed into a more active state after initiation of seed germination in addition to protein and energy-related metabolism. Fourteen DEPs involved in defense response or the oxidation-reduction process were also observed, including myrosinase-binding protein 2 (spots 16, 18, and 19), 1-Cys peroxiredoxin PER1 (spots 826 and 832), peptide methionine sulfoxide reductase B5 (spots 961 and 967), and others, indicating the onset of defense mechanisms during seed germination.

### Hierarchical cluster analysis of DEPs

Hierarchical clustering analysis was performed for all the 113 DEPs, and five cluster patterns were revealed (Patterns I-V; Figure 4). Further analysis showed that Pattern II (29 DEPs) and pattern IV (18 DEPs) represented the abundance-decreased proteins in post-germination seedling growth and germinating seed, respectively. For instance, the expression of three storage proteins (cruciferin 2, 12S seed storage protein CRA1, and 12S seed storage protein CRD) was decreased during seed germination, indicating that these storage proteins might be mobilized and consumed as a nutrient reservoir in germinating seed. Pattern I (16 DEPs) was represented as the DEPs that kept increasing from 18 HAI. A defense-related protein, MBP2, which was involved in metabolizing glucosinolates and formed defense compounds to protect against herbivore attack, exhibited this pattern, implying that the myrosinase-glucosinolate defense system was activated at the beginning of post-germination seedling growth. In addition, pattern III (29 DEPs) and pattern V (17 DEPs) represented proteins that gradually changed during seed germination, and the highest protein abundance was detected at 36 HAI. These proteins reflected several active pathways (e.g., pentose phosphate pathway, glycolysis/gluconeogenesis, protein translation, and protein folding) during seed germination (Figure 4B). Thirty-three out of 113 DEPs fell into patterns I and V, which suggested that a number of DEPs might be *de novo* synthesized upon seed germination (Figure 4).

### Protein-protein interaction among DEPs

**Figure 5:**
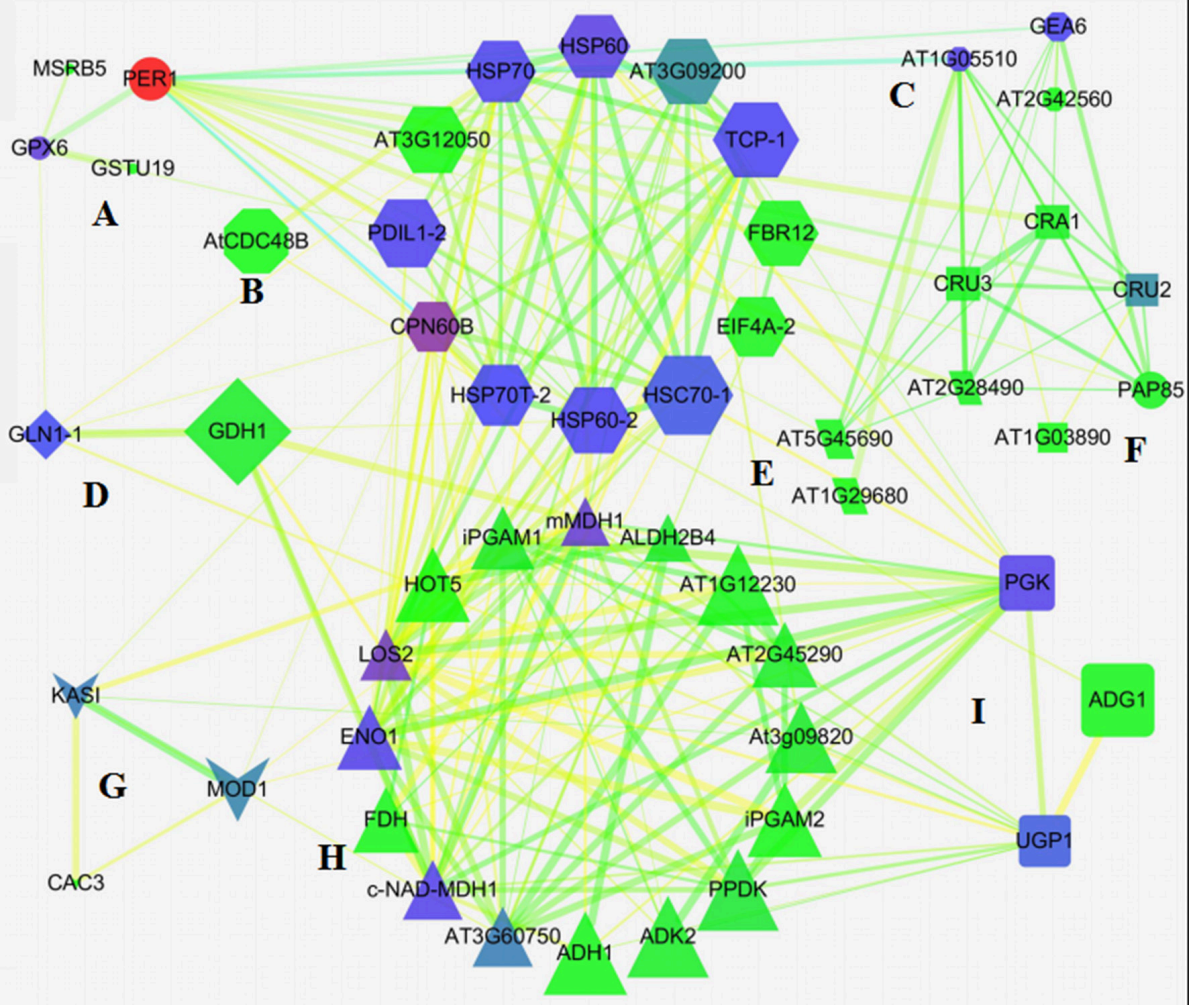
**Network of DEPs in germinating seeds. (A)** Defense/disease; **(B)** unknown; **(C)** seed maturation; **(D)** amino acids metabolism; **(E)** protein metabolism; **(F)** storage proteins; **(G)** lipid metabolism; **(H)** energy metabolism; **(I)** carbohydrate metabolism.

To predict the relationship among the DEPs, protein-protein interaction networks were constructed using STRING 10.0. The 113 DEPs were matched with 68 unique homologs in *Arabidopsis* by BLAST in the NCBI database (Table S3). Out of the 68 proteins, 66 representing 111 DEPs were depicted in the STRING database (Figure S4). The interaction networks were then re-visualized by Network Analyzer. As shown in Figure 5, nine tightly connected clusters were illuminated in the network based on the functional classification (Figure 5), for example, 13 unique homologous proteins were connected with each other in cluster E, and most of them were involved in protein synthesis, processing, and degradation. Besides, many members involved in the oxidation-reduction process, and energy and carbohydrate metabolism also appeared to be closely linked with these proteins, which indicated that redox homeostasis, and energy and carbohydrate metabolism were crucial for activating protein metabolism in germinating seed. Cluster H included multiple enzymes involved in glycolysis, gluconeogenesis, the pentose phosphate pathway, and the TCA cycle, implying the material and energy supply active upon seed germination (Figure 5). These DEPs also were linked with other DEPs involved in protein metabolism, amino metabolism, carbohydrate metabolism, and lipid metabolism, reflecting a crucial role in metabolism as the source of ATP, acetyl-CoA, NADH, and carbon skeleton (Figure 5).

### Construction of the potential metabolism pathway based on DEPs

**Figure 6:**
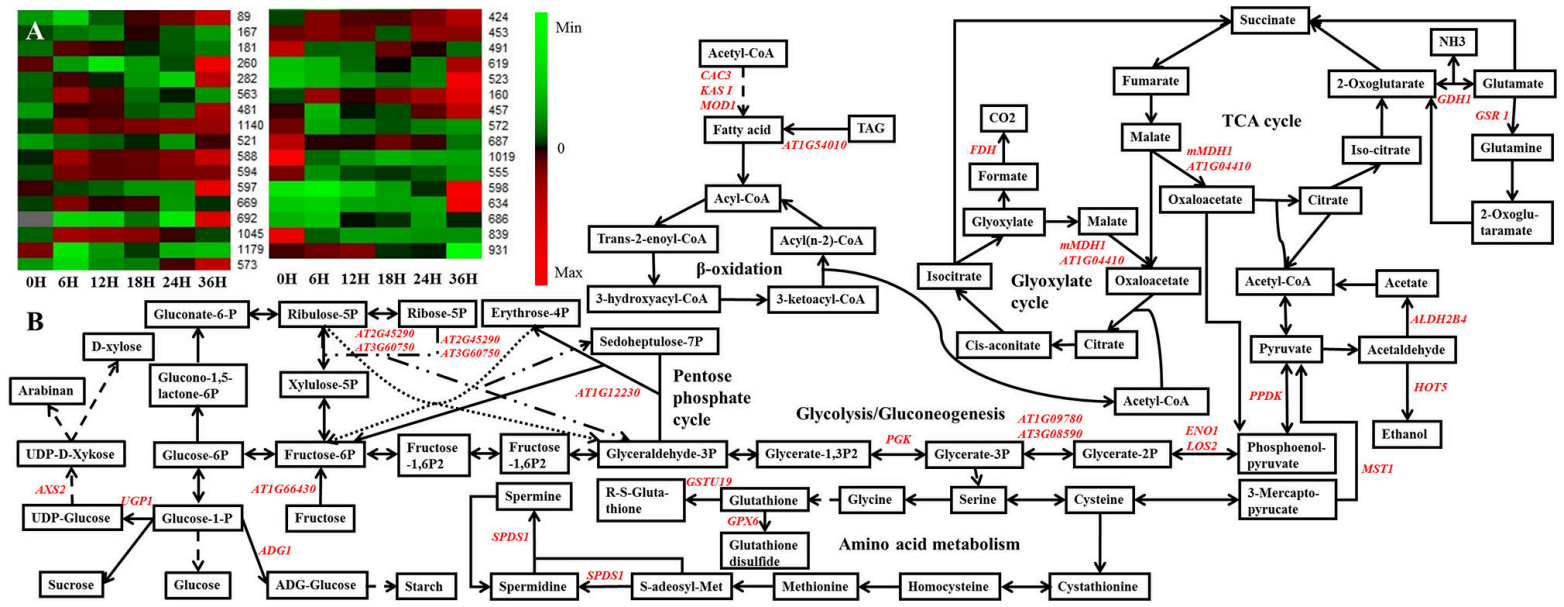
**The metabolic pathways based on the proteomic data. (A)** The expression pattern of DEPs showed in the metabolic pathways. **(B)** the metabolic pathways. Gene symbol and corresponding DEP spot numbers are as follows: *ADG1*, spot 491; *ALDH2B4*, spot 1179; *AT1G04410*, spot 1045; *AT1G09780*, spot 260; *AT1G12230*, spot 597; *AT1G54010*, spot 572; *AT1G66430*, spot 619; *AT2G45290*, spot 167; *AT3G08590*, spot 282; *AT3G60750*, spot 181; *AXS2*, spot 523; *CAC3*, spot 160; *ENO1*, spot 481; *FDH*, spot 573; *GDH 1*, spot 555; *GPX6*, spot 931; *GSR 1*, spot 598; *GSTU19*, spot 839; *HOT5*, spot 521 and 563; *KAS I*, spot 457; *LOS2*, spot 1140; *mMDH1*, spot 669 and 692; *MOD1*, spot 687 and 1019; *MST1*, spot 686; *PGK*, spot 588 and 594; *PPDK*, spot 89; *SPDS1*, spot 634; *UGP1*, spot 424 and 453.

To further analyze the metabolism changes during seed germination, a potential metabolism pathway was constructed based on the DEPs during seed germination of *B. napus* by using the KEGG database (http://www.kegg.jp/) and previous research (Graham, 2008; He et al., 2011; Han et al., 2013; Figure 6). Here, we use a heatmap to show the expression patterns of the 33 DEPs that were constructed into the potential metabolism pathway according to a search of the KEGG database and previous research (Figure 6A; Graham, 2008; He et al., 2011; Han et al., 2013). These DEPs were located in several pathways, mainly involved in the TCA cycle, glyoxylate cycle, glycolysis/gluconeogenesis, pentose phosphate pathway, and amino acid metabolism (Figure 6B). Combination with the expression pattern shown in Figure 6A clearly revealed that the glycolysis/gluconeogenesis, pentose phosphate pathway, TCA cycle, and glyoxylate cycle might be activated earlier than amino acid metabolism during seed germination, while some sulfur amino acid metabolism-related DEPs, which were closely associated with plant redox homeostasis and defense systems (Rajjou et al., 2012), were also activated upon seed germination.

### Mapping identification of the genes corresponding to DEPs

To unravel the relationship between these DEPs and the genetic control of seed germination and vigor, the candidate genes for seed germination and vigor revealed through genome-wide association mapping performed by Hatzig et al. (2015) were used for further analysis. Through searching genes that were detected within and near 20 associated LD regions of six traits related to seed germination and vigor, 17 genes corresponding to 11 DEPs were identified in *B. napus* (Table 3). Among them, DEP AT4G25580, annotated as CAP160 protein, corresponding to *BnaC07g39780D* located near a LD region was associated with volume increase. Five DEPs (corresponding to *BnaA03g15330D, BnaA03g16750D, BnaA03g32320D, BnaA03g33160D*, and *BnaC09g39680D*) that were involved in protein and amino acid metabolism and the glycolysis pathway (Tables 2, 3) and located near three LD regions were associated with GR72 on chromosomes A03 and C09 (2 and 1, respectively) (Table 3). Four genes of three DEPs (APA1, AXS2, and AT1G03890) were observed to be close to a common LD region associated with T50 and GR36 on C08. Of them, the two copies (*BnaC08g43570D* and *BnaC08g43590D*) of *AT1G03890* that encodes the 12S seed storage protein CRD could be found near this region. The other two DEPs (APA1 and AXS2) were identified as corresponding to *BnaA09g47450D* and *BnaA09g48990D*, and were close to another LD region, just associated with T50 on A09. APA1 (aspartic proteinase A1) is involved in proteolysis and response to salt stress during the seedling development stage in *Arabidopsis* (Huttlin et al., 2007; Mazorra-Manzano et al., 2010). AXS2, a UDP-D-apiose/UDP-D-xylose synthase, is involved in amino acid metabolism during the seedling development stage (Giavalisco et al., 2005). Near an ES-related LD region on chromosome A10, three copies (*BnaA10g27060D, BnaA10g27070D*, and *BnaA10g27080D*) of the gene that encodes HSC70-1 (a heat shock cognate protein) were identified. ADK2 (adenosine kinase 2) was also located near this region, and GDH1 (glutamate dehydrogenase 1) was identified adjacent to another LD region associated with ES on chromosome A10.

**Table 3:** **DEPs verified within and near associated LD regions in seed germination and vigor-related genome-wide association mapping**.

**Trait**	**LD region (bp)**	***B. napus* gene**	**Chr^a^**	**Position (bp)**	**Spot No**.	***Ath* locus^b^**	**Gene symbol**	**FC^c^**
GR72	7,082,702–7,098,892	BnaA03g15330D	A03	7,112,072–7,115,439	1080	AT2G33210	HSP60-2	Protein metabolism
		BnaA03g16750D	A03	7,826,124–7,828,960	1140	AT2G36530	LOS2	Glycolysis/Gluconeogenesis
	16,074,818–16,173,009	BnaA03g32320D	A03	15,595,154–15,597,674	213	AT3G12580	HSP70	Protein metabolism
		BnaA03g33160D	A03	16,042,287–16,045,648	483	AT3G13920	EIF4A1	Protein metabolism
	42,980,135–43,015,797	BnaC09g39680D	C09	42,312,710–42,315,089	555	AT5G18170	GDH1	Amino acids metabolism
T50	32,206,450–32,410,348	BnaA09g47450D	A09	31,985,234–31,990,347	676	AT1G11910	APA1	Protein metabolism
		BnaA09g48990D	A09	32,704,420–32,707,246	523	AT1G08200	AXS2	Amino acids metabolism
ES	11,631,861–11,677,068	BnaA10g16660D	A10	12,602,040–12,604,476	555	AT5G18170	GDH1	Amino acids metabolism
	16,085,669–16,164,217	BnaA10g26640D	A10	16,947,590–16,950,180	576	AT5G03300	ADK2	Energy metabolism
		BnaA10g27060D	A10	17,109,978–17,111,513	219	AT5G02500	HSC70-1	Protein metabolism
		BnaA10g27070D	A10	17,114,576–17,115,925	219	AT5G02500	HSC70-1	Protein metabolism
		BnaA10g27080D	A10	17,115,989–17,116,994	219	AT5G02500	HSC70-1	Protein metabolism
VI	41,147,197–41,318,742	BnaC07g39780D	C07	40,476,619–40,478,919	190	AT4G25580	AT4G25580	Unknown
T50/GR36	36,326,419–3,638,4818	BnaC08g41720D	C08	36,216,640–36,220,278	676	AT1G11910	APA1	Protein metabolism
		BnaC08g43300D	C08	37,085,013–37,087,718	523	AT1G08200	AXS2	Amino acids metabolism
		BnaC08g43570D	C08	37,216,210–37,218,152	952	AT1G03890	AT1G03890	Storage proteins
		BnaC08g43590D	C08	37,222,097–37,223,773	952	AT1G03890	AT1G03890	Storage proteins

a Chromosome.

b Arabidopsis Locus.

c Functional classification.

### Expression of genes encoding the key DEPs

**Figure 7:**
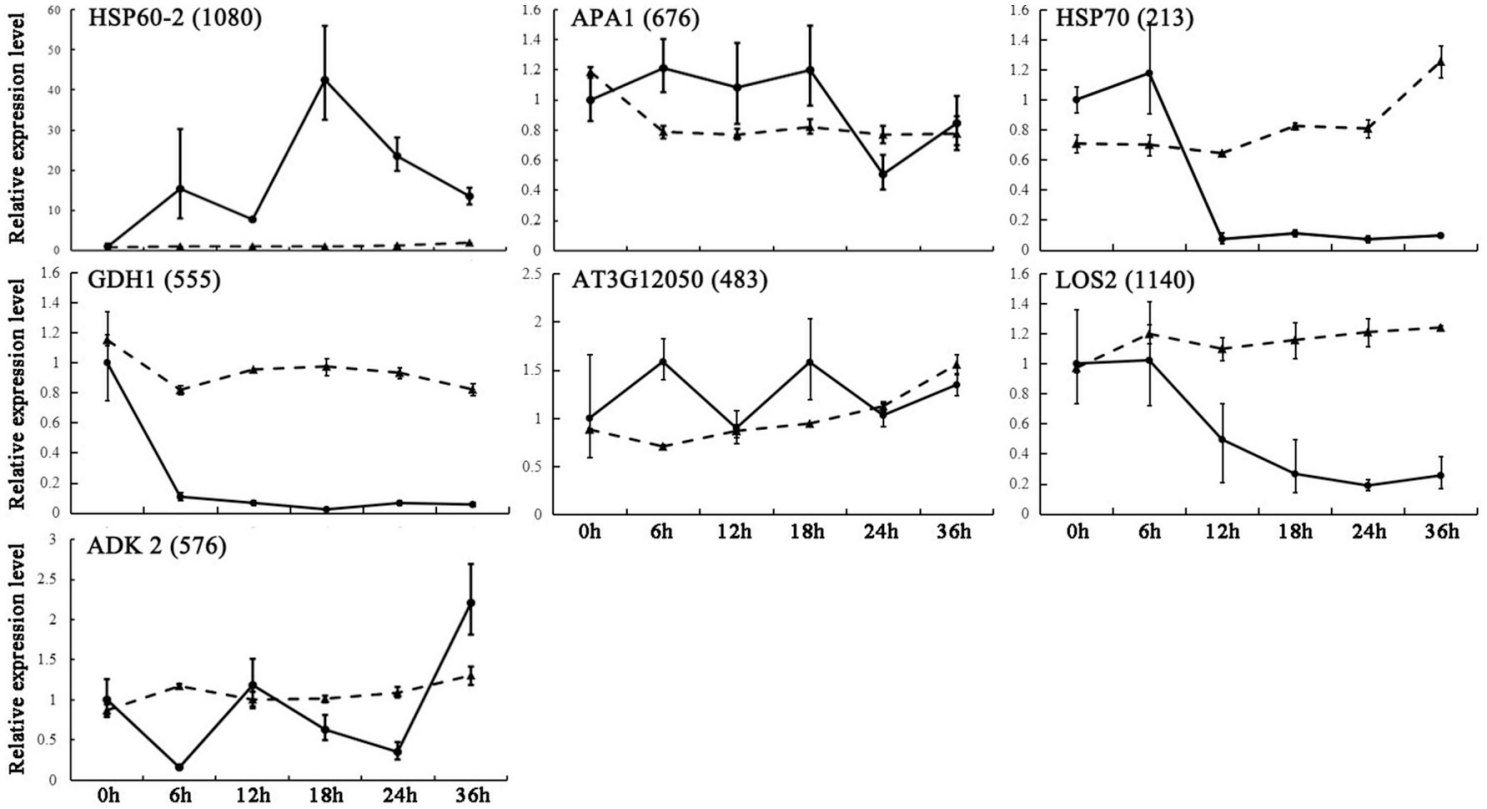
**qRT-PCR analysis of gene expression patterns in germinating seeds**. Relative expression levels of homologous genes of DEPs from *B. napus* seeds were determined by qRT-PCR analysis. The value were determined 0, 6, 12, 18, 24, and 36 HAI and are presented as means ± standard error (*n* = 3). Different lines show relative expression levels of homologous genes (solid line) and corresponding proteins (dotted line).

By combing our proteomic analysis results with genome-wide analysis results reported previously, we found 17 genes corresponding to 11 DEPs located in or near the confidence interval of QTLs for seed germination and vigor. The gene expression upon seed germination was analyzed by quantitative real-time (qRT)-PCR. The present results demonstrated that seven genes showed multiple expression patterns (Figure 7), and these were involved in glycolysis/gluconeogenesis, amino acid metabolism and protein metabolism (Table 2). Among them, glutamate dehydrogenase 1 (*GDH1*, spot 555) appeared to have similar expression patterns to the corresponding DEPs (Figure 7). In addition, three metabolism-related genes and one energy metabolism-related gene, Aha1 domain-containing protein (*AT3G12050*, spot 483), 60S acidic ribosomal protein P0-2 (*APA 1*, spot 676), heat shock protein 60-2 (*HSP60-2*, spot 1080), and adenosine kinase 2 (*ADK2*, spot 576), showed consistent trends of expression with the homologous proteins. Furthermore, gene expression of heat shock protein 70-4 (*HSP70*, spot 213) and bifunctional enolase 2/transcriptional activator (*LOS2*, spot 1140), which are involved in protein folding and energy metabolism, showed opposite trends to the expression of the homologous proteins, respectively (Figure 7). The results indicated that the aforementioned metabolic processes were modulated by post-transcriptional and/or post-translational regulation during seed germination. The inconsistent abundances of transcripts and proteins in germinating seed also supported the notion that pre-synthesized mRNA and proteins in mature dry seeds would function for seed germination (Sano et al., 2012).

## Discussion

### Heterotrophic metabolism is active in germinating seed of *B. napus*

The physical and metabolic events occurring during germination and early seedling growth include respiration, DNA synthesis, transcription and translation of new mRNAs, radicle emergence, mobilization of reserves in storage tissues, and mobilization of major reserves (Graham, 2008; Nonogaki et al., 2010). Heterotrophic metabolism is suspected to contribute to seed germination, and the present TEM results revealed that the predominant reserve organs (OBs and PBs) decreased gradually during seed germination. Similarly, a clear decrease in the number of PBs and OBs after 3 and 4 days of olive (*Olea europaea* L.) cotyledon cell culture was also demonstrated (Zienkiewicz et al., 2011); an obvious decrease of OBs was also observed at 48 h of imbibition in the endosperm cells of germinating *J. curcas* seed (Yang et al., 2009). Consistently, it was observed that oil content remained stable at the early stage of germination and was rapidly consumed during seedling establishment in wild-type *Arabidopsis* WS and Col0 as well as the acyl-CoA oxidase gene mutants *acx1-1, acx2-1*, and *acx1-1acx2-1* (Pinfield-Wells et al., 2005); the present results also showed a similar tendency in *B. napus*. This indicated that seed storage oil mobilization might be not essential for germination but is very important for seedling establishment.

By introduction of fluorescent cyanine dyes and an internal standard, the ability of 2-D DIGE to separate and resolve complex proteomic patterns was greatly improved (Van den Bergh and Arckens, 2004; Rozanas and Loyland, 2008). Although, 2-D DIGE has not totally overcome the shortfalls of gel-based proteomics technology, there is still not a method that can completely replace 2-D DIGE to simultaneously separate and display several thousand proteins from complex samples. Besides these above-mentioned physiology and biochemistry results, 46.9% DEPs involved in energy metabolism, carbohydrate metabolism, protein metabolism, amino acid metabolism, and lipid metabolism were also observed by 2-D DIGE combined with MS and MS/MS and a Mascot database search in *B. napus*. Similarly, 50 DEPs were identified during *J. curcas* seed germination, 34% (17) of which were oil mobilization-related proteins involved in the glyoxylate cycle, glycolysis, the citric acid cycle, gluconeogenesis, and the pentose phosphate pathway (Yang et al., 2009). With the assistance of KEGG analysis and previous research (Graham, 2008; He et al., 2011; Han et al., 2013), a metabolism pathway was constructed containing the DEPs involved during seed germination in *B. napus*. It was revealed that the pentose phosphate pathway (e.g., transketolase and transketolase, spots 167 and 181), TCA cycle (e.g., malate dehydrogenase, spots 669, 692, and 1045), glycometabolism (e.g., pfkB-like carbohydrate kinase family protein, spot 619), amino acid metabolism (e.g., mercaptopyruvate sulfurtransferase 1 and spermidine synthase 1, spots 686 and 634), and fatty acid metabolism (e.g., alcohol dehydrogenase 2B4, spot 1179) were actively altered upon seed germination (Figure 6; Table 2). This indicated that the mobilization and sequential hydrolysis of storage reserves (proteins, starch, and lipids) are tightly controlled temporally and spatially, which is important for energy metabolite biosynthesis during seed germination.

In *B. napus*, the necessary amino acids required for the *de novo* synthesis of proteins after germination is provided by cruciferin and napin (Nykiforuk and Johnson-Flanagan, 1999). The major storage protein, cruciferin, accounts for ~50–60% of the total seed protein (Crouch and Sussex, 1981; Höglund et al., 1992). Cruciferin was shown to be significantly decreased after 48 HAI at 22°C and was no longer detected at 72 HAI in *B. napus* (Nykiforuk and Johnson-Flanagan, 1999). The present results also revealed that the total protein content started to decrease sharply from 36 HAI, and 26 DPEs assigned as storage proteins during seed germination were identified, of which, 16 spots were dramatically decreased after 36 HAI. As storage proteins might have been generated by the initiation of seed storage mobilization, it is not surprising that 10 spots that might have been generated through the breakdown of storage proteins were significantly increased at 36 HAI (Table S2). Consistent with previous studies (Nykiforuk and Johnson-Flanagan, 1999; Fu et al., 2005; Yang et al., 2009), our results showed that heterotrophic metabolism is active in germinating *B. napus* seed in the dark. Storage sugar and protein, as the main energy and metabolite sources during *B. napus* seed germination *sensu stricto*, and the consumption of storage oil and protein are essential for seedling establishment.

### The maturation program can be recapitulated during early stages of germination

Maturation usually happens during seed formation; however, Lopez-Molina et al. (2001) proposed that the formation of embryos also arrested in their progress toward germination based on the known function of the genes controlled by ABI3 during seed maturation, including those encoding LEA proteins. Seed germination of *Arabidopsis* was also shown to reset the maturation program, as the *de novo* synthesis of LEA proteins and storage proteins (cruciferins) was observed (Rajjou et al., 2004). The possibility of recapitulating the late maturation program at the transcriptional and translational levels would allow rapid adjustment of the response of imbibed seeds confronted with rapid fluctuations in environmental conditions (Lopez-Molina et al., 2001; Rajjou et al., 2004, 2006). A seed maturation-related protein (spot 1025, uncharacterized protein, corresponding gene: *AT1G05510*) induced during the *B. napus* seed germination was also observed; this showed that the maturation program might also be recapitulated during early stages of seed germination in *B. napus*.

### Establishment of defense systems during seed germination in *B. napus*

It is well-known that plants have devised sophisticated mechanisms to cope with biotic and abiotic stresses imposed by their environment. In the plant life cycle, the seed and seedling stages are key developmental stages that are sensitive to stresses (Bewley and Black, 1994; Koornneef et al., 2002). The present results revealed that the expression of some sulfur amino acid metabolism-related proteins was increased during seed germination in *B. napus*. In both plants and animals, glutathione S-transferases are induced by diverse environmental stimuli and play direct roles in reducing oxidative damage or toxic products produced during xenobiotic metabolism (Dixon et al., 2002; Moons, 2005; Frova, 2006; Xu et al., 2016). The glutathione S-transferase TAU 19 (GSTU19; spot 839) was observed to have a relatively high expression level even at 36 HAI that was decreased upon seed germination. Glutathione peroxidases (spot 931) are a group of enzymes that protect cells against oxidative damage generated by reactive oxygen species, and play an important role in conversion of glutathione and glutathione disulfide, which is critical for the reduction of H_2_O_2_, organic hydroperoxides, and lipid peroxides (Sugimoto and Sakamoto, 1997; Milla et al., 2003); the expression of this enzyme was induced after imbibition but started to be repressed at 18 HAI. Methionine sulfoxide reductase B5 (spots 961 and 967) can reverse the reaction of methionine oxidation to methionine sulfoxide, which results in modification of activity and conformation for many proteins (Rouhier et al., 2006); these spots showed high expression levels during germination and post-germination growth and all were decreased at 36 HAI. The sulfur amino acid metabolism pathway represents a determinant biochemical key of the commitment of the seed to initiate its development toward germination (Rajjou et al., 2012), and inhibition of sulfur amino acid metabolism can strongly delay seed germination and seedling growth (Gallardo et al., 2002; Bassel et al., 2008; Fulneček et al., 2011). Methionine is a fundamental metabolite among the essential amino acids synthesized by plants; it does not only function as a building block for protein synthesis but also as the precursor of S-adenosylmethionine, the universal methyl-group donor, and the precursor of polyamines, ethylene, and the vitamin biotin (Ranocha et al., 2001). In *Arabidopsis*, some other sulfur amino acid metabolism-related proteins, such as methionine synthase and S-adenosylmethionine synthase, also showed accumulation at different stages of seed germination (Gallardo et al., 2001, 2002; Rajjou et al., 2012). These results highlight the important role of sulfur amino acid metabolism during seed germination.

Besides the sulfur amino acid metabolism pathway-related proteins, the myrosinase-glucosinolate system also is involved in plant development and defense, and could affect the behavior of herbivorous insects and pathogens (Rask et al., 2000; Capella et al., 2001). Myrosinase-binding proteins were identified as components of high-molecular-mass myrosinase complexes in extracts of *B. napus* seeds (Lenman et al., 1990; Falk et al., 1995; Taipalensuu et al., 1997). It was also shown that complex formation of myrosinase isoenzymes in oilseed rape seeds was dependent on the presence of myrosinase-binding proteins (Eriksson et al., 2002). Consistent with the results showed by Geshi and Brandt (1998), four myrosinase-binding protein spots were shown to be increased during seed germination and were highly expressed at 36 HAI. This implied the onset of defense mechanisms at the level of seed germination.

## Author contributions

JG carried out the proteomic analysis and wrote the manuscript and Lu Gan participant in the proteomic experiments. HC built DEPs identification through the results of the genome-wide associated mapping and completed the manuscript. Liangxing Guo, NR, and KZ made helpful suggestions to the manuscript and participated in the qRT-PCR experiments. HW and YL provided plant materials. ML designed, led, and coordinated the overall study.

### Conflict of interest statement

The authors declare that the research was conducted in the absence of any commercial or financial relationships that could be construed as a potential conflict of interest.

## Supplementary material

Table S1 **Primer design for genes encoding key DEPs involved in seed germination**.

Table S2 **Protein expression patterns of DEPs during germination**. 0H, 6H, 12H, 18H, 24H, and 36H represent the 0, 6, 12, 18, 24, and 36 HAI.

Table S3 **The corresponding unique genes of DEPs**. The unique genes that matched with each DEPs were searched by blast in NCBI database. ^a^chrome; ^b^the gi number of each deferentially expressed protein in the NCBI database; ^c^the name representing each DEPs in STRING.

Table S4 **The peptide matches of DEPs**. Peptide sequences identified by MS-MS sequencing and corresponding to DEPs in *B. napus* seeds identified in this work. % Cov, coverage.

Figure S1 **The content of each fatty acid and total fatty acids determined during seed germination. (A)** The change of total fatty acid content during *B. napus* seed germination; **(B,C)** The change of each fatty acid content during *B. napus* seed germination.

Figure S2 **The significantly differently expressed DEPs during seed germination and seedling establishment**. 6 h/0 h, 12 h/6 h, 18 h/12 h, 24 h/18 h, and 36 h/24 h represent the comparison among different germination stages.

Figure S3 **The protein-protein interaction network in germinating seed revealed by STRING analysis**. The name of each node is the same as the STRING symbol in Table S3.

Figure S4 **The replicate DIGE gels of different germination stages**. Green color shows that the sample was labeled by cy3. Red color shows the sample was labeled by cy5.

## References

[B1] Bassel G. W. Fung P. Chow T. F. Foong J. A. Provart N. J. Cutler S. R.. (2008). Elucidating the germination transcriptional program using small molecules. Plant Physiol. 147, 143–155. 10.1104/pp.107.110841 18359847 PMC2330302

[B2] Bassel G. W. Lan H. Glaab E. Gibbs D. J. Gerjets T. Krasnogor N.. (2011). Genome-wide network model capturing seed germination reveals coordinated regulation of plant cellular phase transitions. Proc. Natl. Acad. Sci. U.S.A. 108, 9709–9714. 10.1073/pnas.1100958108 21593420 PMC3111290

[B3] Benincasa P. Pace R. Quinet M. Lutts S. (2013). Effect of salinity and priming on seedling growth in rapeseed (*Brassica napus var oleifera Del*.). Acta Sci. Agron. 35, 479–486. 10.4025/actasciagron.v35i4.17655

[B4] Bewley J. D. (1997). Seed germination and dormancy. Plant Cell. 9:1055. 10.1105/tpc.9.7.1055 12237375 PMC156979

[B5] Bewley J. D. Black M. (1994). Seeds: Physiology of Development and Germination. New York, NY: Springer US Press 10.1007/978-1-4899-1002-8

[B6] Browse J. McCourt P. J. Somerville C. R. (1986). Fatty acid composition of leaf lipids determined after combined digestion and fatty acid methyl ester formation from fresh tissue. Anal Biochem. 152, 141–145. 10.1016/0003-2697(86)90132-6 3954036

[B7] Bybordi A. Tabatabaei J. (2009). Effect of salinity stress on germination and seedling properties in canola cultivars (*Brassica napus* L.). Not. Bot. Hort. Agrobot. 37, 71 10.15835/nbha3723299

[B8] Capella A. N. Menossi M. Arruda P. Benedetti C. E. (2001). COI1 affects myrosinase activity and controls the expression of two flower-specific myrosinase-binding protein homologues in *Arabidopsis*. Planta 213, 691–699. 10.1007/s004250100548 11678272

[B9] Crouch M. L. Sussex I. M. (1981). Development and storage-protein synthesis in *Brassica napus* L. *embryos in vivo* and in vitro. Planta 153, 64–74. 10.1007/BF00385319 24276708

[B10] Dixon R. A. Achnine L. Kota P. Liu C. J. Reddy M. S. Wang L. (2002). The phenylpropanoid pathway and plant defence-a genomics perspective. Mol. Plant Pathol. 3, 371–390. 10.1046/j.1364-3703.2002.00131.x 20569344

[B11] Eriksson S. Andréasson E. Ekbom B. Granér G. Pontoppidan B. Taipalensuu J.. (2002). Complex formation of myrosinase isoenzymes in oilseed rape seeds are dependent on the presence of myrosinase-binding proteins. Plant Physiol. 129, 1592–1599. 10.1104/pp.003285 12177471 PMC166746

[B12] Falk A. Taipalensuu J. Ek B. Lenman M. Rask L. (1995). Characterization of rapeseed myrosinase-binding protein. Planta 195, 387–395. 10.1007/BF00202596 7766044

[B13] Frova C. (2006). Glutathione transferases in the genomics era: new insights and perspectives. Biomol. Eng. 23, 149–169. 10.1016/j.bioeng.2006.05.020 16839810

[B14] Fu Q. Wang B. C. Jin X. Li H. B. Han P. Wei K. H.. (2005). Proteomic analysis and extensive protein identification from dry, germinating *Arabidopsis* seeds and young seedlings. J. Biochem. Mol. Biol. 38:650. 10.5483/BMBRep.2005.38.6.650 16336779

[B15] Fulneček J. Matyášek R. Votruba I. Holý A. Křížová K. Kovařík A. (2011). Inhibition of SAH-hydrolase activity during seed germination leads to deregulation of flowering genes and altered flower morphology in tobacco. Mol. Genet. Genomics 285, 225–236. 10.1007/s00438-011-0601-8 21274566

[B16] Gallardo K. Job C. Groot S. P. Puype M. Demol H. Vandekerckhove J.. (2001). Proteomic analysis of *Arabidopsis* seed germination and priming. Plant Physiol. 126, 835–848. 10.1104/pp.126.2.835 11402211 PMC111173

[B17] Gallardo K. Job C. Groot S. P. Puype M. Demol H. Vandekerckhove J.. (2002). Proteomics of *Arabidopsis* seed germination. A comparative study of wild-type and gibberellin-deficient seeds. Plant Physiol. 129, 823–837. 10.1104/pp.002816 12068122 PMC161704

[B18] Gan L. Zhang C. Y. Wang X. D. Wang H. Long Y. Yin Y. T.. (2013). Proteomic and comparative genomic analysis of two *Brassica napus* lines differing in oil content. J. Proteome Res. 12, 4965–4978. 10.1021/pr4005635 24053668

[B19] Ge F. Hu Y. Wang J. (2013). Spatial and temporal gene expression during seed germination of *Brassica napus*. Acta Physiol. Plant. 35, 2939–2950. 10.1007/s11738-013-1324-8

[B20] Ge F. W. Tao P. Zhang Y. Wang J. B. (2014). Characterization of *AQP* gene expressions in *Brassica napus* during seed germination and in response to abiotic stresses. Biol. Plant. 58, 274–282. 10.1007/s10535-013-0386-1

[B21] Geshi N. Brandt A. (1998). Two jasmonate-inducible myrosinase-binding proteins from *Brassica napus* L. seedlings with homology to jacalin. Planta 204, 295–304. 10.1007/s004250050259 9530873

[B22] Giavalisco P. Nordhoff E. Kreitler T. Klöppel K. D. Lehrach H. Klose J.. (2005). Proteome analysis of *Arabidopsis thaliana* by two-dimensional gel electrophoresis and matrix-assisted laser desorption/ionisation-time of flight mass spectrometry. Proteomics 5, 1902–1913. 10.1002/pmic.200401062 15815986

[B23] Graham I. A. (2008). Seed storage oil mobilization. Annu. Rev. Plant Biol. 59, 115–142. 10.1146/annurev.arplant.59.032607.092938 18444898

[B24] Guo B. Chen Y. Zhang G. Xing J. Hu Z. Feng W.. (2013). Comparative proteomic analysis of embryos between a maize hybrid and its parental lines during early stages of seed germination. PLoS ONE 8:e65867. 10.1371/journal.pone.0065867 23776561 PMC3679168

[B25] Hajduch M. Casteel J. E. Hurrelmeyer K. E. Song Z. Agrawal G. K. Thelen J. J. (2006). Proteomic analysis of seed filling in *Brassica napus*. Developmental characterization of metabolic isozymes using high-resolution two-dimensional gel ecectrophoresis. Plant Physiol. 141, 32–46. 10.1104/pp.105.075390 16543413 PMC1459325

[B26] Han C. Yin X. He D. Yang P. (2013). Analysis of proteome profile in germinating soybean seed, and its comparison with rice showing the styles of reserves mobilization in different crops. PLoS ONE 8:e56947. 10.1371/journal.pone.0056947 23460823 PMC3584108

[B27] Hatzig S. Zaharia L. I. Abrams S. Hohmann M. Legoahec L. Bouchereau A.. (2014). Early osmotic adjustment responses in drought-resistant and drought-sensitive oilseed rape. J. Integr. Plant Biol. 56, 797–809. 10.1111/jipb.12199 24667002

[B28] Hatzig S. V. Frisch M. Breuer F. Nesi N. Ducournau S. Wagner M. H.. (2015). Genome-wide association mapping unravels the genetic control of seed germination and vigor in *Brassica napus*. Front. Plant Sci. 6:221. 10.3389/fpls.2015.00221 25914704 PMC4391041

[B29] He D. Han C. Yao J. Shen S. Yang P. (2011). Constructing the metabolic and regulatory pathways in germinating rice seeds through proteomic approach. Proteomics 11, 2693–2713. 10.1002/pmic.201000598 21630451

[B30] Höglund A. S. Rödin J. Larsson E. Rask L. (1992). Distribution of napin and cruciferin in developing rape seed embryos. Plant Physiol. 98, 509–515. 10.1104/pp.98.2.509 16668669 PMC1080218

[B31] Huttlin E. L. Hegeman A. D. Harms A. C. Sussman M. R. (2007). Comparison of full versus partial metabolic labeling for quantitative proteomics analysis in *Arabidopsis thaliana*. Mol. Cell. Proteomics 6, 860–881. 10.1074/mcp.M600347-MCP200 17293592

[B32] Janmohammadi M. Fallahnezhad F. Golsha M. Mohammadi H. (2008). Controlled ageing for storability assessment and predicting seedling early growth of canola cultivars (*Brassica napus* L.). ARPN J. Agric. Biol. Sci. 3, 22–26.

[B33] Katayama H. Nagasu T. Oda Y. (2001). Improvement of in-gel digestion protocol for peptide mass fingerprinting by matrix-assisted laser desorption/ionization time-of-flight mass spectrometry. Rapid Commun. Mass Spectrom. 15, 1416–1421. 10.1002/rcm.379 11507753

[B34] Kelly A. A. Quettier A. L. Shaw E. Eastmond P. J. (2011). Seed storage oil mobilization is important but not essential for germination or seedling establishment in *Arabidopsis*. Plant Physiol. 157, 866–875. 10.1104/pp.111.181784 21825108 PMC3192569

[B35] Kondra Z. P. Campbell D. C. King J. R. (1983). Temperature effects on germination of rapeseed (*Brassica napus* L. and *B. campestris* L.). Can. J. Plant Sci. 63, 1063–1065.

[B36] Koornneef M. Bentsink L. Hilhorst H. (2002). Seed dormancy and germination. Curr. Opin. Plant. Biol. 5, 33–36. 10.1016/S1369-5266(01)00219-9 11788305

[B37] Kruger N. J. (2009). The Bradford method for protein quantitation, in The Protein Protocols Handbook, ed Walker J. M. (New York, NY: Humana Press), 17–24. 10.1007/978-1-59745-198-7_4

[B38] Kubala S. Garnczarska M. Wojtyla Ł. Clippe A. Kosmala A. Żmieńko A.. (2015). Deciphering priming-induced improvement of rapeseed (*Brassica napus* L.) germination through an integrated transcriptomic and proteomic approach. Plant Sci. 231, 94–113. 10.1016/j.plantsci.2014.11.008 25575995

[B39] Lenman M. Rödin J. Josefsson L. G. and Rask, L. (1990). Immunological characterization of rapeseed myrosinase. Eur. J. Biochem. 194, 747–753. 10.1111/j.1432-1033.1990.tb19465.x 2269297

[B40] Li F. Wu X. Tsang E. Cutler A. J. (2005). Transcriptional profiling of imbibed *Brassica napus* seed. Genomics 86, 718–730. 10.1016/j.ygeno.2005.07.006 16125897

[B41] Li G. McVetty P. B. (2013). Genetics and gene mapping of disease resistance in *Brassica*, in Translational Genomics for Crop Breeding: Biotic Stress, Vol. 1, eds Varshney R. K. Tuberosa R. (New York, NY: John Wiley & Sons, Inc.), 327–344. 10.1002/9781118728475.ch16

[B42] Liu A. Gao F. Kanno Y. Jordan M. C. Kamiya Y. Seo M.. (2013). Regulation of wheat seed dormancy by after-ripening is mediated by specific transcriptional switches that induce changes in seed hormone metabolism and signaling. PLoS ONE 8:e56570. 10.1371/journal.pone.0056570 23437172 PMC3577873

[B43] Long Y. Shi J. Qiu D. Li R. Zhang C. Wang J.. (2007). Flowering time quantitative trait loci analysis of oilseed *Brassica* in multiple environments and genomewide alignment with *Arabidopsis*. Genetics 177, 2433–2444. 10.1534/genetics.107.080705 18073439 PMC2219480

[B44] Lopez-Molina L. Mongrand S. Chua N. H. (2001). A postgermination developmental arrest checkpoint is mediated by abscisic acid and requires the ABI5 transcription factor in *Arabidopsis*. Proc. Natl. Acad. Sci. U.S.A. 98, 4782–4787. 10.1073/pnas.081594298 11287670 PMC31911

[B45] Mazorra-Manzano M. A. Tanaka T. Dee D. R. Yada R. Y. (2010). Structure-function characterization of the recombinant aspartic proteinase A1 from Arabidopsis thaliana. Phytochemistry 71, 515–523. 10.1016/j.phytochem.2009.12.005 20079503

[B46] Milla M. A. R. Maurer A. Huete A. R. Gustafson J. P. (2003). Glutathione peroxidase genes in Arabidopsis are ubiquitous and regulated by abiotic stresses through diverse signaling pathways. Plant J. 36, 602–615. 10.1046/j.1365-313X.2003.01901.x 14617062

[B47] Mohammadi G. R. (2009). The influence of NaCl priming on seed germination and seedling growth of canola (*Brassica napus* L.) under salinity conditions. Am. Euras. J. Agric. Environ. Sci. 5, 696–700.

[B48] Moons A. (2005). Regulatory and functional interactions of plant growth regulators and plant glutathione S-transferases (GSTs). Vitam. Horm. 72, 155–202. 10.1016/S0083-6729(05)72005-7 16492471

[B49] Müller K. Tintelnot S. Leubner-Metzger G. (2006). Endosperm-limited *Brassicaceae* seed germination: abscisic acid inhibits embryoinduced endosperm weakening of *Lepidium sativum* (cress) and endosperm rupture of cress and *Arabidopsis thaliana*. Plant Cell Physiol. 47, 864–877. 10.1093/pcp/pcj059 16705010

[B50] Nagel M. Rosenhauer M. Willner E. Snowdon R. J. Friedt W. Börner A. (2011). Seed longevity in oilseed rape (*Brassica napus* L.)–genetic variation and QTL mapping. Plant Genet. Resour. 9, 260–263. 10.1017/S1479262111000372

[B51] Nguyen T. C. Obermeier C. Friedt W. Abrams S. R. Snowdon R. J. (2016). Disruption of germination and seedling development in *Brassica napus* by mutations causing severe seed hormonal imbalance. Front. Plant Sci. 7:322. 10.3389/fpls.2016.00322 27014334 PMC4791391

[B52] Nonogaki H. Bassel G. W. Bewley J. D. (2010). Germination—still a mystery. Plant Sci. 179, 574–581. 10.1016/j.plantsci.2010.02.010

[B53] Nykiforuk C. L. Johnson-Flanagan A. M. (1999). Storage reserve mobilization during low temperature germination and early seedling growth in *Brassica napus*. Plant Physiol. Biochem. 37, 939–947. 10.1016/S0981-9428(99)00108-4

[B54] Obermeier C. Hossain M. A. Snowdon R. Knüfer J. von Tiedemann A. Friedt W. (2013). Genetic analysis of phenylpropanoid metabolites associated with resistance against *Verticillium longisporum* in *Brassica napus*. Mol. Breed. 31, 347–361. 10.1007/s11032-012-9794-8

[B55] Osman K. A. Tang B. Wang Y. Chen J. Yu F. Li L.. (2013). Dynamic QTL analysis and candidate gene mapping for waterlogging tolerance at maize seedling stage. PLoS ONE 8:e79305. 10.1371/journal.pone.0079305 24244474 PMC3828346

[B56] Pfaffl M. W. Tichopad A. Prgomet C. Neuvians T. P. (2004). Determination of stable housekeeping genes, differentially regulated target genes and sample integrity: BestKeeper–Excel-based tool using pair-wise correlations. Biotechnol Lett. 26, 509–515. 10.1023/B:BILE.0000019559.84305.47 15127793

[B57] Pinfield-Wells H. Rylott E. L. Gilday A. D. Graham S. Job K. Larson T. R.. (2005). Sucrose rescues seedling establishment but not germination of *Arabidopsis* mutants disrupted in peroxisomal fatty acid catabolism. Plant J. 43, 861–872. 10.1111/j.1365-313X.2005.02498.x 16146525

[B58] Pritchard S. L. Charlton W. L. Baker A. Graham I. A. (2002). Germination and storage reserve mobilization are regulated independently in *Arabidopsis*. Plant J. 31, 639–647. 10.1046/j.1365-313X.2002.01376.x 12207653

[B59] Rajjou L. Belghazi M. Huguet R. Robin C. Moreau A. Job C.. (2006). Proteomic investigation of the effect of salicylic acid on *Arabidopsis* seed germination and establishment of early defense mechanisms. Plant Physiol. 141, 910–923. 10.1104/pp.106.082057 16679420 PMC1489900

[B60] Rajjou L. Duval M. Gallardo K. Catusse J. Bally J. Job C.. (2012). Seed germination and vigor. Annu. Rev. Plant Biol. 63, 507–533. 10.1146/annurev-arplant-042811-105550 22136565

[B61] Rajjou L. Gallardo K. Debeaujon I. Vandekerckhove J. Job C. Job D. (2004). The effect of α-amanitin on the *Arabidopsis* seed proteome highlights the distinct roles of stored and neosynthesized mRNAs during germination. Plant Physiol. 134, 1598–1613. 10.1104/pp.103.036293 15047896 PMC419834

[B62] Ranocha P. McNeil S. D. Ziemak M. J. Li C. Tarczynski M. C. Hanson A. D. (2001). The S-methylmethionine cycle in angiosperms: ubiquity, antiquity and activity. Plant J. 25, 575–584. 10.1046/j.1365-313x.2001.00988.x 11309147

[B63] Rask L. Andréasson E. Ekbom B. Eriksson S. Pontoppidan B. Meijer J. (2000). Myrosinase: gene family evolution and herbivore defense in *Brassicaceae*, in Plant Molecular Evolution, eds Doyle J. J. Gaut B. S. (Dordrecht: Springer Netherlands Press), 93–113. 10.1007/978-94-011-4221-2_5 10688132

[B64] Reyes J. L. Chua N. H. (2007). ABA induction of miR159 controls transcript levels of two MYB factors during *Arabidopsis* seed germination. Plant J. 49, 592–606. 10.1111/j.1365-313X.2006.02980.x 17217461

[B65] Rouhier N. Vieira Dos Santos C. Tarrago L. Rey P. (2006). Plant methionine sulfoxide reductase A and B multigenic families. Photosynth. Res. 89, 247–262. 10.1007/s11120-006-9097-1 17031545

[B66] Rozanas C. R. Loyland S. M. (2008). Capabilities using 2-D DIGE in proteomics research, in Tissue Proteomics, Vol. 441, eds Liu B. C.-S. Ehrlich J. R. (New York, NY: Humana Press), 1–18. 10.1007/978-1-60327-047-2_1 18370308

[B67] Rücker B. Röbbelen G. (1996). Impact of low linolenic acid content on seed yield of winter oilseed rape (*Brassica napus* L.). Plant Breed. 115, 226–230. 10.1111/j.1439-0523.1996.tb00908.x

[B68] Sano N. Permana H. Kumada R. Shinozaki Y. Tanabata T. Yamada T.. (2012). Proteomic analysis of embryonic proteins synthesized from long-lived mRNAs during germination of rice seeds. Plant Cell Physiol. 53, 687–698. 10.1093/pcp/pcs024 22383627

[B69] Schiessl S. Samans B. Hüttel B. Reinhard R. Snowdon R. J. (2014). Capturing sequence variation among flowering-time regulatory gene homologs in the allopolyploid crop species *Brassica napus*. Front. Plant Sci. 5:404. 10.3389/fpls.2014.00404 25202314 PMC4142343

[B70] Schopfer P. Plachy C. (1985). Control of Seed Germination by Abscisic Acid III. Effect on Embryo Growth Potential (Minimum Turgor Pressure) and Growth Coefficient (Cell Wall Extensibility) in *Brassica napus* L. Plant Physiol. 77, 676–686. 10.1104/pp.77.3.676 16664118 PMC1064584

[B71] Shu L. Lou Q. Ma C. Ding W. Zhou J. Wu J.. (2011). Genetic, proteomic and metabolic analysis of the regulation of energy storage in rice seedlings in response to drought. Proteomics 11, 4122–4138. 10.1002/pmic.201000485 21818852

[B72] Sreenivasulu N. Usadel B. Winter A. Radchuk V. Scholz U. Stein N.. (2008). Barley grain maturation and germination: metabolicpathway and regulatory network commonalities and differences highlighted by new MapMan/PageMan Profiling Tools. Plant Physiol. 146, 1738–1758. 10.1104/pp.107.111781 18281415 PMC2287347

[B73] Srivastava S. Fristensky B. Kav N. N. (2004). Constitutive expression of a PR10 protein enhances the germination of *Brassica napus* under saline conditions. Plant Cell Physiol. 45, 1320–1324. 10.1093/pcp/pch137 15509856

[B74] Sugimoto M. Sakamoto W. (1997). Putative phospholipid hydroperoxide glutathione peroxidase gene from *Arabidopsis thaliana* induced by oxidative stress. Genes Genet. Syst. 72, 311–316. 10.1266/ggs.72.311 9511228

[B75] Taipalensuu J. Eriksson S. Rask L. (1997). The Myrosinase-binding protein from *Brassica napus* seeds possesses lectin activity and has a highly similar vegetatively expressed wound-inducible counterpart. Eur. J. Biochem. 250, 680–688. 10.1111/j.1432-1033.1997.00680.x 9461290

[B76] Tang W. Deng Z. Oses-Prieto J. A. Suzuki N. Zhu S. Zhang. (2008). Proteomics studies of brassinosteroid signal transduction using prefractionation and two-dimensional DIGE. Mol. Cell. Proteomics 7, 728–738. 10.1074/mcp.M700358-MCP200 18182375 PMC2401332

[B77] Tsadilas C. D. Shaheen S. M. (2013). Utilization of biosolids in production of bioenergy crops II: impact of application rate on bioavailability and uptake of trace elements by canola. Commun. Soil Sci. Plant Anal. 44, 259–274. 10.1080/00103624.2013.741773

[B78] Van den Bergh G. Arckens L. (2004). Fluorescent two-dimensional difference gel electrophoresis unveils the potential of gel-based proteomics. Curr. Opin. Biotech. 15, 38–43. 10.1016/j.copbio.2003.12.001 15102464

[B79] Wang H. Z. Yin Y. (2014). Analysis and strategy for oil crop industry in China. Chin. J. Oil Crop Sci. 36, 414–421. 10.7505/j.issn.1007-9084.2014.03.020

[B80] Wei F. Yi J. W. Wang X. F. Dong X. Y. Li P. P. Li Y. H. (2009). A quick method for oil contents determination in small quantity of rape seed. Chin. J. Oil Crop Sci. 31, 517–521. 10.3321/j.issn:1007-9084.2009.04.021

[B81] Wen C.-f. Dong A.-w. Li G.-z. Lei S. Lei Y. (2005). Determination of total sugar and reducing sugar in *Viola Philippica* ssp. Munda W. Becker by anthrone colorimetry. Mod. Food Sci. Technol. 21, 122–124. 10.3969/j.issn.1673-9078.2005.03.044

[B82] Xu J. Tian Y. S. Xing X. J. Peng R. H. Zhu B. Gao J. J. (2016). Over-expression of *AtGSTU19* provides tolerance to salt, drought and methyl viologen stresses in *Arabidopsis*. Physiol. Plant. 156, 164–175. 10.1111/ppl.12347 25975461

[B83] Xu X. Y. Fan R. Zheng R. Li C. M. Yu D. Y. (2011). Proteomic analysis of seed germination under salt stress in soybeans. J. Zhejiang Univ. Sci. B 12, 507–517. 10.1631/jzus.B1100061 21726057 PMC3134839

[B84] Yang M. F. Liu Y. J. Liu Y. Chen H. Chen F. Shen S. H. (2009). Proteomic analysis of oil mobilization in seed germination and postgermination development of *Jatropha curcas*. J. Proteome Res. 8, 1441–1451. 10.1021/pr800799s 19152324

[B85] Yang P. Li X. Wang X. Chen H. Chen F. Shen S. (2007). Proteomic analysis of rice (*Oryza sativa*) seeds during germination. Proteomics 7, 3358–3368. 10.1002/pmic.200700207 17849412

[B86] Zhang J. Jiang F. Yang P. Li J. Yan G. Hu L. (2015). Responses of canola (*Brassica napus* L.) cultivars under contrasting temperature regimes during early seedling growth stage as revealed by multiple physiological criteria. Acta Physiol. Plant. 37, 1–10. 10.1007/s11738-014-1748-9

[B87] Zhang X. K. Yang G. T. Chen L. Yin J. M. Tang Z. L. Li J. N. (2006). Physiological differences between yellow-seeded and black-seeded rapeseed (*Brassica napus* L.) with different testa characteristics during artificial ageing. Seed Sci. Technol. 34, 373–381. 10.15258/sst.2006.34.2.13

[B88] Zhang Y. Chen B. Xu Z. Shi Z. Chen S. Huang X.. (2014). Involvement of reactive oxygen species in endosperm cap weakening and embryo elongation growth during lettuce seed germination. J. Exp. Bot. 65, 3189–3200. 10.1093/jxb/eru167 24744430 PMC4071836

[B89] Zheng G. H. Wilen R. W. Slinkard A. E. Gusta L. V. (1994). Enhancement of canola seed germination and seedling emergence at low temperature by priming. Crop Sci. 34, 1589–1593. 10.2135/cropsci1994.0011183X003400060031x

[B90] Zienkiewicz A. Jiménez-López J. C. Zienkiewicz K. de Dios Alché J. Rodríguez-García M. I. (2011). Development of the cotyledon cells during olive (*Olea europaea* L.) *in vitro* seed germination and seedling growth. Protoplasma 248, 751–765. 10.1007/s00709-010-0242-5 21104420

