# Supplementary figures and images for: Proteomic Dissection of Seed Germination and Seedling Establishment in Brassica napus
Source: Front Plant Sci. 2016 Oct 24;7:1482. doi: 10.3389/fpls.2016.01482 (PMC5075573; doi:10.3389/fpls.2016.01482)

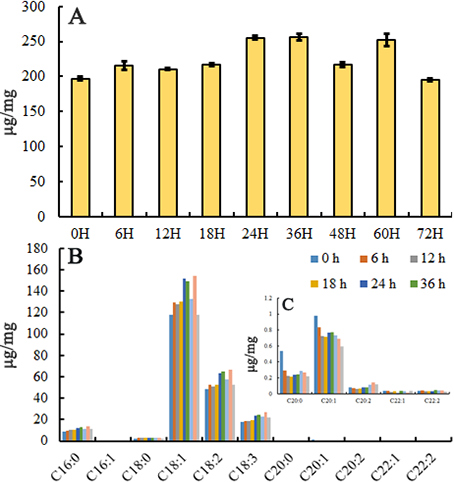

Supplement: Figure S1 — The content of each fatty acid and total fatty acids determined during seed germination. (A) The change of total fatty acid content during B. napus seed germination; (B,C) The change of each fatty acid content during B. napus seed germination. [file Image1.TIF]

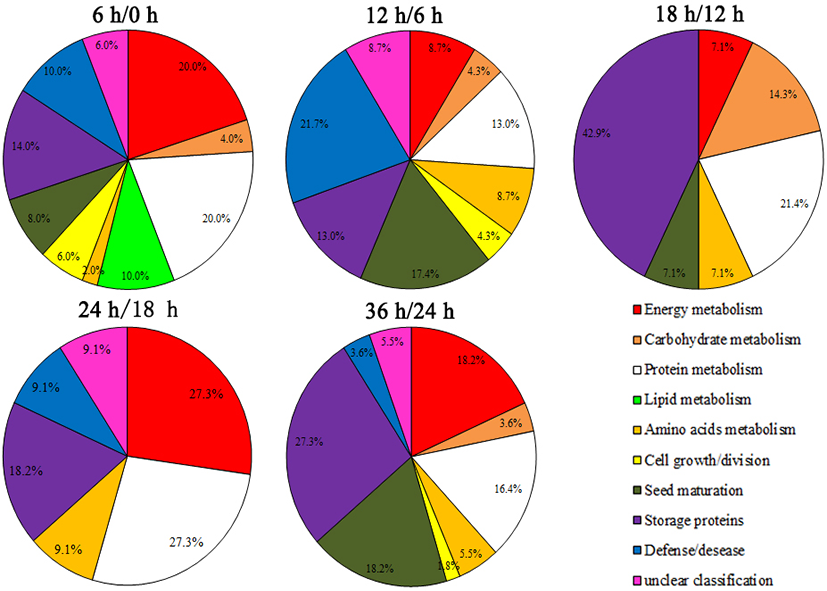

Supplement: Figure S2 — The significantly differently expressed DEPs during seed germination and seedling establishment. 6 h/0 h, 12 h/6 h, 18 h/12 h, 24 h/18 h, and 36 h/24 h represent the comparison among different germination stages. [file Image2.TIF]

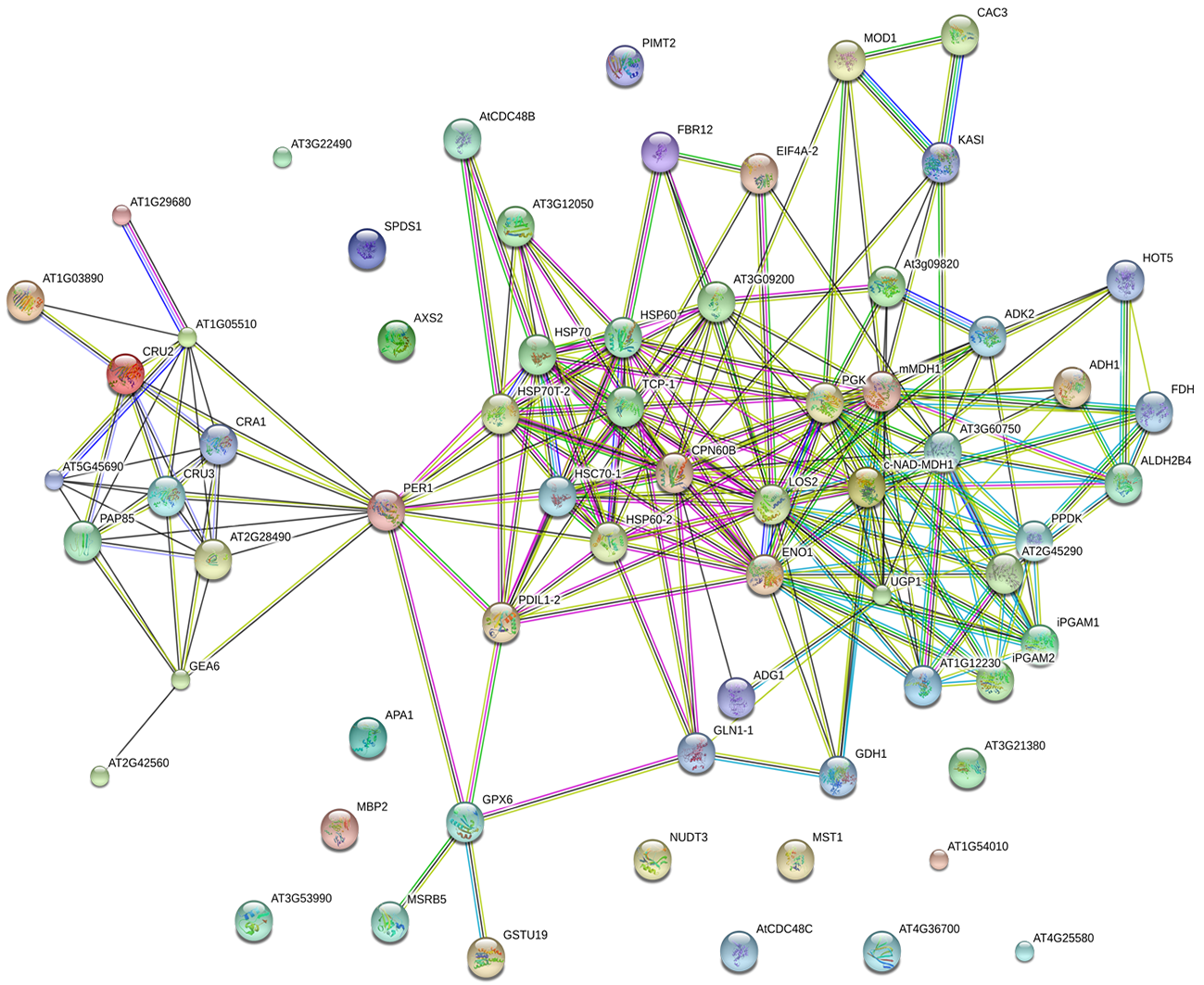

Supplement: Figure S3 — The protein-protein interaction network in germinating seed revealed by STRING analysis. The name of each node is the same as the STRING symbol in Table S3. [file Image3.TIF]

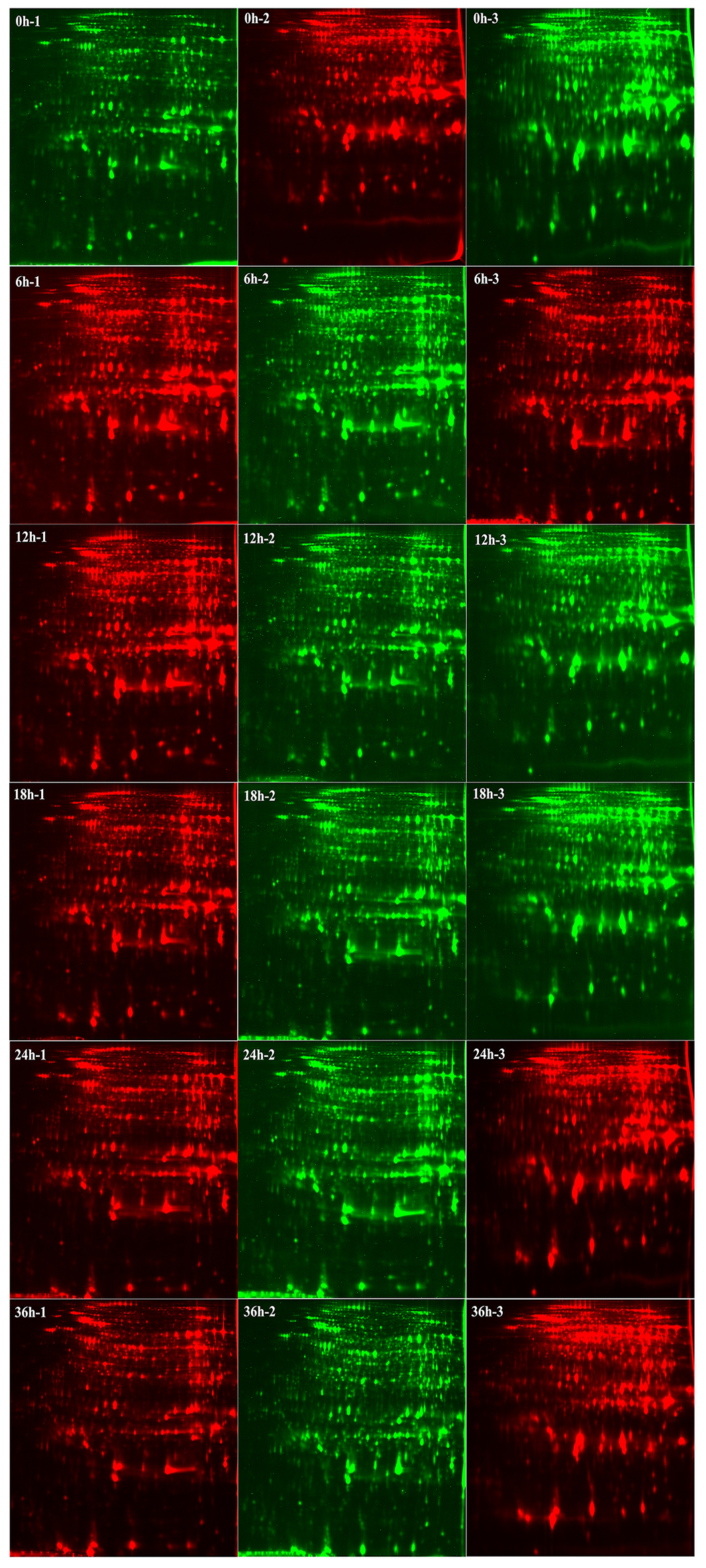

Supplement: Figure S4 — The replicate DIGE gels of different germination stages. Green color shows that the sample was labeled by cy3. Red color shows the sample was labeled by cy5. [file Image4.TIF]
